# On the Mechanism of Soft Self‐Assembly from Melt: The Ubiquitous Heat Capacity Hump and Spontaneous Melt Chirality

**DOI:** 10.1002/anie.202505548

**Published:** 2025-05-15

**Authors:** Yi‐nan Xue, Xiang‐bing Zeng, Bo‐wen Wu, Ya‐xin Li, Liliana Cseh, Shu‐gui Yang, Jie Liu, Gillian A. Gehring, Feng Liu, Goran Ungar

**Affiliations:** ^1^ Shaanxi International Research Center for Soft Matter State Key Laboratory for Mechanical Behaviour of Materials Xi'an Jiaotong University Xi'an 710049 China; ^2^ School of Chemical Materials and Biological Engineering University of Sheffield Sheffield S1 3JD UK; ^3^ School of Chemistry and Chemical Engineering Henan University of Technology Zhengzhou 450001 China; ^4^ Romanian Academy Coriolan Dragulescu Institute of Chemistry Timisoara 300223 Romania; ^5^ School of Mathematical and Physical Sciences University of Sheffield Sheffield S3 7RH UK

**Keywords:** 2D Ising model, Aggregation, Bicontinuous, Liquid crystals, Polycatenar

## Abstract

We investigate two unusual phenomena in self‐assembly of anisotropic molecules from isotropic (Iso) melt: a heat‐capacity (*C*_p_) maximum and spontaneous formation of the recently discovered chiral liquid (Iso*). Based on experiments on new nonchiral monomers, dimers, and polymers, we construct a statistical theory that shows why many complex meso‐structures form in two stages: continuous equilibrium growth of nano‐clusters in melt through strong interactions, causing the *C*_p_‐maximum, followed by establishment of positional long‐range order (LRO) through a weak first‐order transition. We also show why many achiral compounds additionally form an intermediate chiral Iso* liquid through what we find is a second‐order transition. We propose that the first process is equivalent to “supramolecular polymerization” in solutions, where the lack of intercluster interaction rules out LRO. Furthermore, we argue that separation into a broad and a sharp transition is universal in condensed matter where strong interactions by themselves cannot lead to LRO, either because the clusters are 1D or due to strong frustration. Clusters must first grow to critical size when, at *T*_c_, the combined weak interactions reach ∼k_B_ *T*_c_, prompting LRO formation. A situation similar to that in soft self‐assembly is seen in spin ordering in magnetic crystals, but only near 0 K.

## Graphical abstract

Phase transition from melt to most complex liquid crystal (LC) phases is weak 1st order but is preceded by a large heat capacity maximum. In addition, a chiral liquid forms through another transition before that to the LC, despite the compounds being achiral. We investigate these poorly understood phenomena experimentally and develop a statistical theory that fits the experiments remarkably well, giving new insights into the mechanism of self‐assembly and pre‐assembly.

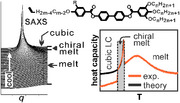

## Introduction

Self‐assembly shapes biological and synthetic systems and gives them functions essential for life and technology. Unraveling the mechanism of formation of complex meso‐structures from the melt usually relies on simulations^[^ ^1^ ^]^ since only the final structure is seen experimentally. Here we show that self‐assembly from melt most often happens in several identifiable thermodynamically stable stages. Consider, e.g., the highly complex 3D structures such as multinetwork bicontinuous cubic or noncubic phases,^[^ ^2^ ^]^ or other lattices of supramolecular objects.^[^ ^3^ ^]^ It is remarkable that the entropy of their phase transition from isotropic melt is minute, on average only half that of the transition from isotropic to nematic liquid that has no positional order—see Table S1 in Supporting Information (SI). So where is the entropy lost and where does the bulk of assembly really happen?

It has been noticed by a few authors in the past^[^ ^4^, ^5^ ^]^ that there exists a broad maximum in specific heat (*C*_p_) in the isotropic (Iso) melt above the transition to a bicontinuous cubic mesophase with long‐range order (LRO). We now investigate this *C*_p_ hump on a variety of newly synthesized compounds, including monomers, dimers, and polymers, and find that above the transition, they all show a *C*_p_ hump, irrespective of whether the ensuing LC phase is bicontinuous cubic, bicontinuous noncubic, columnar, smectic, or even just crystal. It is evident that the phenomenon is widespread and surprisingly poorly studied. It is also clear that this is not a normal pre‐transitional effect as the *C*_p_ decreases rather than increases on approach to the transition. Moreover, the hump can appear even where there is no associated transition. The observations made on our compounds are also compared with other instances, apparently unrelated, where similar *C*_p_ anomalies in melt have been reported. It turns out that all systems involved have one thing in common: simultaneous presence of strong and much weaker interactions. We conclude that whilst strong interactions cause continuous growth of equilibrium clusters, by themselves they cannot lead to LRO; hence, the system remains liquid. To form an ordered structure, they need the weak interactions which, however, are only able to enforce LRO and trigger the transition once the equilibrium clusters reach a critical size.

We explain the *C*_p_ hump quantitatively by developing a thermodynamic theory initially based on Onsager's treatment of magnetic crystals at low temperature featuring strongly anisotropic spin interactions. The theory gives very good quantitative fit to the experimental *C*_p_ profiles. The derived best‐fit parameters then provide quantitative insight into the so‐far untapped process of LC self‐assembly in melt. Moreover, the model also reproduces well the associated spontaneous chirality development in nonchiral compounds and the recently discovered phase transition from normal (Iso) to chiral (Iso*) liquid preceding the establishment of positional LRO.

## Results and Discussion

### Compounds in this Work and Their Thermograms

**Figure 1:**
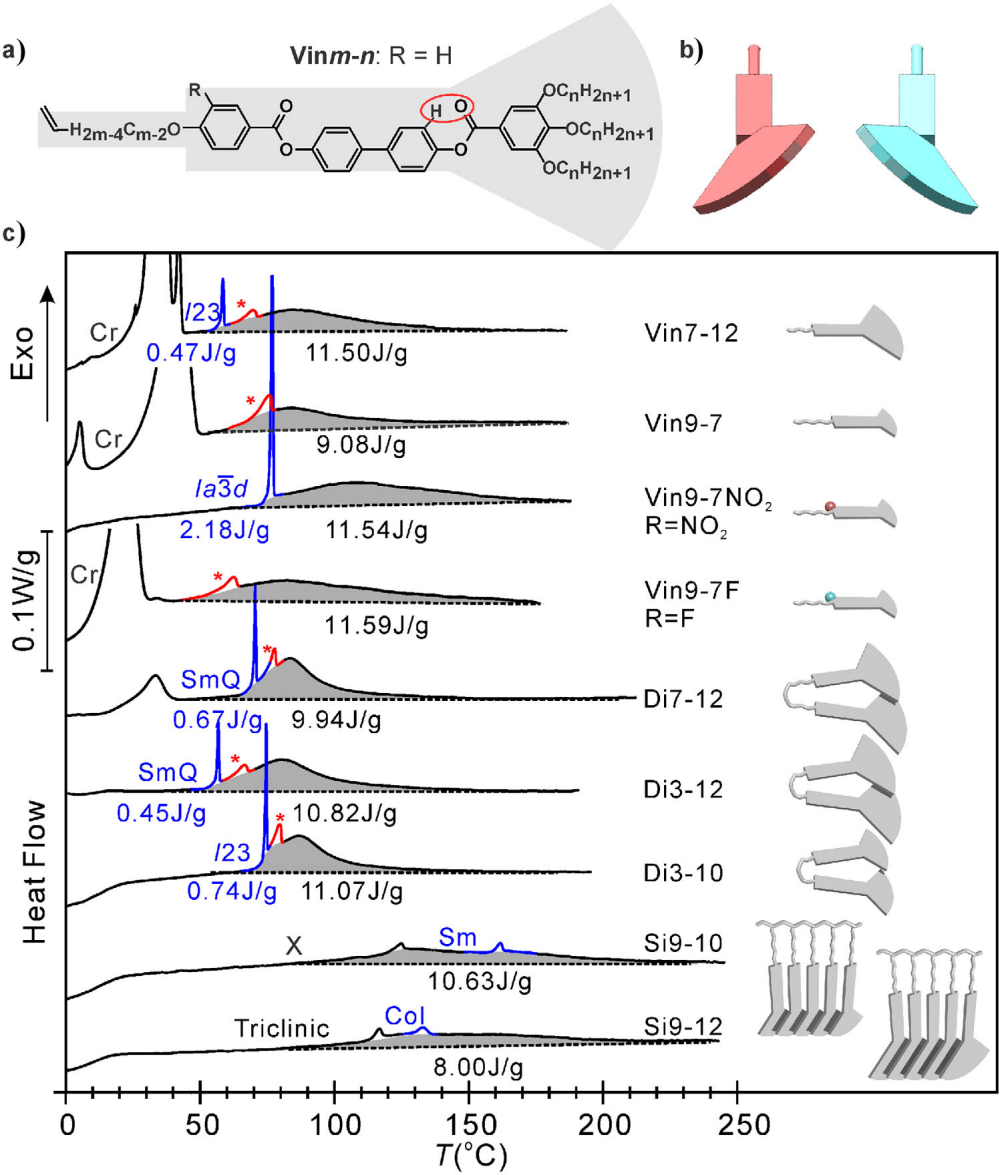
a) General chemical formula of Vin*m‐n* monomers. b) Stylized shape of two enantiomeric conformations of a monomer. c) Cooling DSC thermograms at 5 K min^−1^ of monomers (Vin*m‐n*), dimers (Di*m‐n*), and polymers (Si*m‐n*). Schematic outlines of the molecules are depicted on the right, indicating the variation in size of the tri‐alkoxy fan (*n*) and the tail/spacer (*m*) and, by a small colored ball, the position of the polar substituent (F or NO_2_), where present. High‐*T* phase is Iso liquid; * indicates chiral Iso* liquid. LC phases: SmQ = double‐network tetragonal “Smectic‐Q,” *I*23 = triple‐network cubic, $Ia\bar{3}d$ = double‐network gyroid cubic, Sm = smectic‐A, Col = hexagonal columnar, triclinic = a recent phase of counter‐twisted double helices, and *Y* is as yet unidentified. Cr = crystal. The structure of the LC phases is shown in Figure 2a–f. The peak colored red is the Iso–Iso* transition and that colored blue marks the transition to the first LC phase. Enthalpies under *C*_p_ hump + Iso–Iso* (shaded) are shown in black, and those under the exotherm to LC in blue.

A series of new monomers, dimers, and polymers has been synthesized. The basic unit, the Vin*m*‐*n* monomer, contains a 4‐ring aromatic rod‐like core (the “mesogen”) terminated by three flexible alkyl chains *n* carbons long on a phenyl ring (the “fan”) and with an ω‐alkenyl chain *m* carbons long at the other end (the “spacer”)—see Figure 1a. The compounds are typical “polycatenar” (multichain) LC building blocks.^[^ ^6^, ^7^ ^]^ The dimers Di*m*‐*n* have two such monomers joined via a disiloxane unit, while in the polymers Si*m*‐*n* they are attached to a poly(methylsiloxane) backbone. A stylized monomer which, by the way, has no chiral center, is shown in Figure 1b in two interconvertible enantiomeric conformations, and the shapes of all molecules in this work, with their differences, are depicted schematically on the right in Figure 1c. In order to investigate the effect of substituent groups, in two monomers the R hydrogen is replaced by an electron‐withdrawing group, either nitro (Vin9‐7NO_2_) or a fluorine (Vin9‐7F). The exact chemical structures are shown in Section 9 (Supporting Information), where details of synthesis and chemical characterization can also be found. Polymer Si9‐12 has been synthesized recently, and the structure of its LC phases studied.^[^ ^8^ ^]^ Experimentally, the self‐assembly of the current compounds is studied using optical microscopy, conventional and modulated differential scanning calorimetry (DSC and MDSC), small‐angle X‐ray scattering (SAXS), circular dichroism (CD) spectroscopy, and optical rotation measurement. Details are given in Section 8 (Supporting Information).

Figure 1c shows the cooling DSC thermograms of the compounds. As can be seen, the thermograms all show a *C*_p_ hump within the melt range, seen as a broad exotherm (shaded) above the phase transition to one of the LC states (the sharp exotherms colored blue). The enthalpies contained in the hump and the LC transition are listed in joules per gram below the thermograms. The *C*_p_ hump is attributed to molecular pre‐assembly in the Iso melt. Remarkably, as can be seen, it contains on average ca. 95% of the entropy of assembly, the transition itself being left with only the final ∼5%.

The width of the *C*_p_ hump in Figure 1c varies widely, from the narrowest in Di3‐10 (full‐width‐at‐half‐height FWHH ∼20 K) to the broad and shallow ones in polymers Si9‐10 and Si9‐12 (FWHH ∼60 K). The broad exotherm is also present where crystallization proceeds directly from melt (Vin9‐7, Vin9‐7F). For all monomers and dimers, the peak is centered almost at the same temperature of 80–90 °C, while in polymers this is significantly higher, around 140 °C. Interestingly, despite the diversity of compound structure, the integral excess enthalpies under the broad hump are similar, all between 8.0 and 11.5 J g^−1^. This suggests that the bulk of the enthalpy release comes from aggregation of the aromatic mesogens, which is one thing all compounds have in common. It should, however, be mentioned that the length of the pendant alkyl chains also affects the enthalpy of the hump.^[^ ^4^ ^]^ Note also the significant effect of fluorine and nitro groups in Vin9‐7F and Vin9‐7NO_2_ on broadening the *C*_p_ maximum. Our explanation will be given in Section ‘Interpretation of Experimental Results.’

### The Ordered Phases

**Figure 2:**
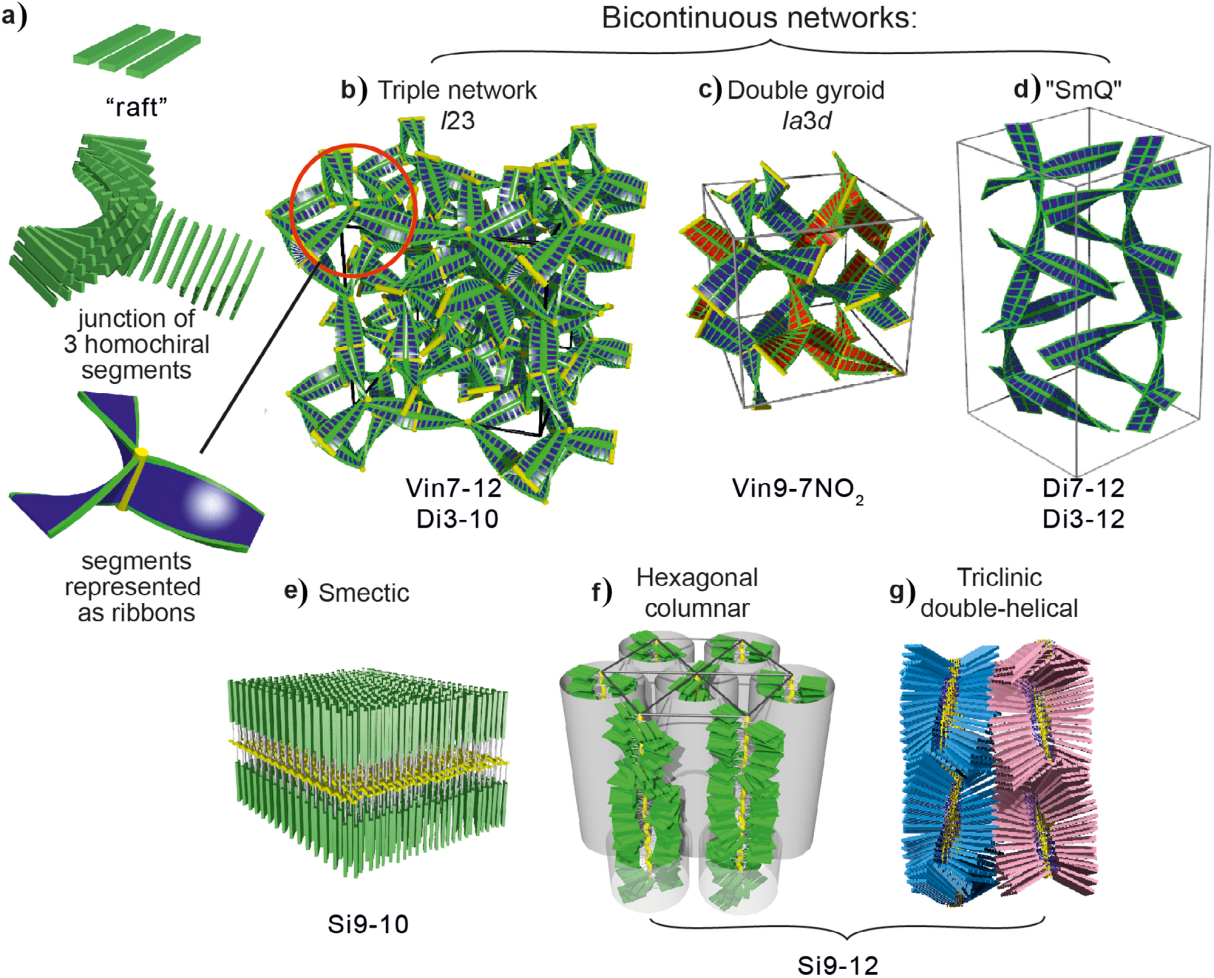
a–c) Twisted ribbon representation of the three network‐based bicontinuous LC phases in the current compounds: (a) triple‐network cubic *I*23 (chiral),^[^ ^9^ ^]^ (b) double‐network gyroid cubic $Ia\bar{3}d$ (achiral),^[^ ^10^, ^11^ ^]^ and (c) double‐network Smectic‐Q *I*4_1_22 (chiral).^[^ ^12^ ^]^ Each green rib in the ribbons represents a raft of 2–4 molecules lying parallel to the rib. The inset on the left shows a raft of 3 molecules and a junction connecting three isochiral network segments represented as molecular rod‐like cores and as twisted ribbons.^[^ ^9^ ^]^ d) Smectic‐A phase (a double‐layer). e) Hexagonal columnar phase. f) Triclinic phase containing antichiral double helices.^[^ ^8^ ^]^ In e–g), the polysiloxane backbone bundles are shown in yellow. The compounds exhibiting the phases are listed below each model.

The LC phases that form through the first‐order transition in our compounds (blue exotherms in Figure 1c) are shown in Figure 2. The nonpolymers form three bicontinuous phases consisting of infinite interpenetrating networks of mesogens: the double‐network “gyroid” cubic, space group $Ia\bar{3}d$,^[^ ^2^, ^10^ ^]^ the triple‐network cubic,^[^ ^13^ ^]^ space group *I*23,^[^ ^9^ ^]^ and the double‐network tetragonal “Smectic‐Q,” space group *I*4_1_22,^[^ ^12^ ^]^ (Figure 2a–c). An alternative to the triple network structure has also been proposed.^[^ ^14^ ^]^ Today we know that these phases,^[^ ^11^ ^]^ as well as some recently discovered columnar types,^[^ ^15^, ^16^ ^]^ contain chiral ribbons formed from stacked “rafts” of 2–4 parallel molecules each—see inset in Figure 2a and further below. The rafts are successively rotated by a relatively small angle (10 ± 3)°, alleviating repulsion between the bulky end‐chains while maximizing the strong aromatic‐core π–π interaction.^[^ ^15^ ^]^ In Figures 2a–c, the phase structures are represented as networks of twisted ribbons, each rib on the ribbon representing a raft with the molecules parallel to the rib. In reality, the rafts are not meant to be flat, distinct species, as sketched schematically in this and subsequent figures. The idea of such slow‐twisting ribbons came after it was realized that the triple network phase is always optically active, whether or not it contains chiral molecules.^[^ ^11^ ^]^ Similarly, it is now known that the SmQ, being always optically active, contains two isochiral networks.^[^ ^12^ ^]^ The third phase, the well‐known gyroid $Ia\bar{3}d$, is achiral because in its two networks the twist sense is opposite, except in strongly chiral compounds where the helicity of one of the networks can invert.^[^ ^17^ ^]^ In all these phases, the long‐range chirality of the networks is maintained by molecular close‐packing in the junctions, requiring that all adjoining ribbons be isochiral (see inset in Figure 2a).

The smectic in Si9‐10 contains double layers of side groups with the polymer backbones sandwiched between these sub‐layers (Figure 2d). The columnar phase in polymer Si9‐12 can be thought of as consisting of straight bundles of polymer backbones on a 2D hexagonal lattice, each bundle surrounded by a sheath of side groups^[^ ^6^, ^8^ ^]^ (Figure 2e). In the triclinic LC phase below, the columns transform into antichiral squashed double helices.^[^ ^8^ ^]^ Optical micrographs of these two phases are shown in Figures S1 and S2 (Supporting Information). X‐ray evidence of all LC phases is shown in Figure S3 and Tables S2–S9. The smectic and columnar nature of the LC phases below the hump in the polymers suggests that the *C*_p_ anomaly may accompany any LC phase except nematic.

The small exothermic peak colored red in Figure 1c, seen in all monomers and dimers except Vin9‐7NO_2_, is the transition from normal Iso liquid to the chiral liquid Iso*, to be discussed further below.

### *C*_p_ Hump in Other Systems

**Figure 3:**
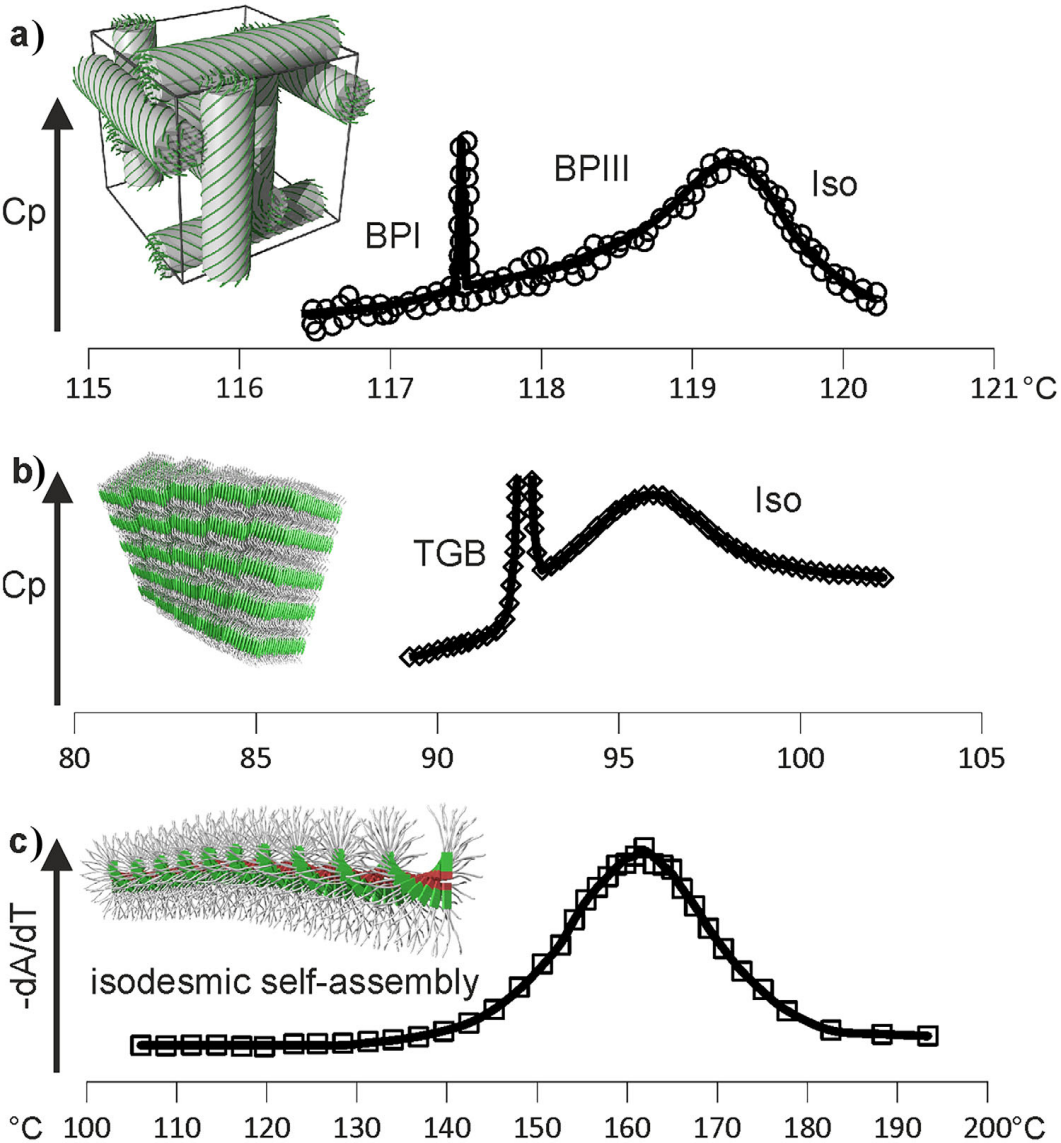
Soft phases of different types and their *C*_p_(*T*) curves in other systems exhibiting a pronounced *C*_p_ maximum in their Iso melt: a) Formation of the disordered “blue‐fog” phase (BPIII) from Iso (DSC curve redrawn after Ref. ^[^ ^19^ ^]^). b) A large *C*_p_ maximum in a chiral mesogen prior to the formation of the “twist grain boundary” (TGB) and SmC* phases on cooling from Iso (DSC curve redrawn after Ref. ^[^ ^28^ ^]^). c) Progress of isodesmic self‐assembly of helical aggregates in solution, monitored by the inverse gradient of UV absorbance at *λ* = 490 nm as a function of temperature; the broad transition band is similar to the *C*_p_ hump in thermotropic melts (calculated from data in Ref. ^[^ ^29^ ^]^).

The appearance of a broad *C*_p_ hump in the melt above the temperature of the transition to a LRO phase is not unique to the present and closely related systems. It appears to be a widespread phenomenon. As already mentioned, a *C*_p_ hump was noted above the two then‐known bicontinuous cubic phases already in the 1990s.^[^ ^4^, ^18^ ^]^ The *C*_p_ anomaly was referred to as “Iso1‐Iso2”^[^ ^4^ ^]^ or, later, as “Iso‐to‐cybotactic Iso” transition.^[^ ^5^ ^]^ Other examples of a diffuse *C*_p_ maximum above the true transition are illustrated in Figure 3.

**Figure 4:**
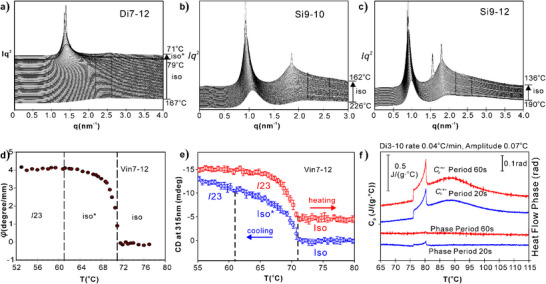
a–c) Pronounced narrowing of the diffuse SAXS maxima as Di7‐12, Si9‐10, and Si9‐12 are cooled through the *C*_p_ hump and (in (a)) the Iso–Iso* transition, before sharp Bragg peaks of the LC phases appear. d) Optical rotation angle *φ* of Vin7‐12 on cooling from Iso melt through Iso* to the cubic *I*23 phase. e) Circular dichroism at 315 nm of a thin film of Vin7‐12; a small amount (1 mol%) of S811 chiral dopant^[^ ^46^ ^]^ was added to the achiral compound in order to bias the spontaneous chirality of the whole sample in the same direction. Heating and cooling rates were 1 K min^−1^. The heating data were shifted up by 5 mdeg for clarity. f) MDSC cooling runs of Di3‐10 at 0.04 K min^−1^, oscillation amplitude 0.07 K, and oscillation period 20 s (blue) and 60 s (red). The top curves show reversing heat capacity *C*_p_^rev^ with the heat flow phase delay angle shown at the bottom.

In some chiral LCs, the remarkable “blue‐fog‐phase III” (BP‐III)^[^ ^19^, ^20^, ^21^ ^]^ has no long‐range order of any kind and is in fact not separate from the normal isotropic liquid, even though the two phases are “separated” by a broad *C*_p_ maximum—see Figure 3a. While the two ordered blue phases I and II have intense color due to monochromatic light diffracted on their regular helices, the “fog” of BPIII comes from diffuse light scattering on its irregular helical domains.^[^ ^22^ ^]^ This is equivalent to the diffuse X‐ray scattering on helical clusters described here in the Iso liquid in our compounds (Figure 4a–c), but having a pitch 100 times longer.

A similar *C*_p_ hump in Iso phase was also found above the twist‐grain‐boundary (TGB) smectic phases (both TGBA and TGBC) in highly chiral mesogens (see Figure 3b), where the isotropic liquid state below the hump was named “L‐phase.”^[^ ^23^, ^24^, ^25^ ^]^ This term was also used for the Iso phase in chiral compounds observed above the Smectic‐Q phase, the structure of which was unknown at the time.^[^ ^26^, ^27^ ^]^

In all the above cases, the bulk of the entropy loss through self‐assembly happens within the broad *C*_p_ hump in the melt with, in most cases, only a marginal final drop at the actual phase transition. While in isolation, the above phenomena have been noted previously, the link between them is missing.

Another “missing link” in the field of self‐assembly is that between assembly from melt (i.e., in the bulk) and the formation of fibrous and helical aggregates from dilute solution. The latter is usually referred to as “supramolecular polymerization.” These isodesmic or weakly cooperative equilibrium self‐assembly processes^[^ ^29^, ^30^, ^31^, ^32^, ^33^, ^34^ ^]^ are also characterized by a *C*_p_ maximum^[^ ^29^ ^]^ although, because of the low DSC signal, the thermograms are usually of poor quality and highly noisy (see Figure 3A in Ref.^[^ ^29^ ^]^) Instead, in Figure 3c, we present the derivative of the cumulative degree of assembly (equivalent to degree of crystallinity) measured by UV spectroscopy,^[^ ^29^ ^]^ which should be proportional to *C*_p_. As normally there cannot be long‐range order in dilute solutions, the process lacks the sharp transition at a lower temperature. In comparison, in “dry” thermotropic systems the equivalent isodesmic process in the melt is only a prelude to the eventual transition to LRO. Notably, the assembly processes in solution have also been compared to living polymerization, similarly displaying a *C*_p_ maximum.^[^ ^35^ ^]^

It should be mentioned that a *C*_p_ maximum has also been observed in liquid tertiary alcohols.^[^ ^36^, ^37^ ^]^ In alcohols, like in water, molecules associate through hydrogen bonding at lower *T* and dissociate at higher *T*, which contributes to their *C*_p_. However, in hindered tertiary alcohols, the OH group is limited to forming only small oligomers, so the association part of the *C*_p_ drops to zero on cooling as there are no more oligomers to form and the existing ones cannot continue growing. These cases are quite special and fundamentally different from those discussed here, since they do not lead to the formation of ordered structures. They can be placed in the category of “self‐limited aggregation,”^[^ ^38^ ^]^ some other examples being helically twisted bundles of fibrous entities^[^ ^39^, ^40^ ^]^ or crystallization of thin layers on curved surfaces.^[^ ^41^ ^]^

### Chiral Isotropic Liquid

Adding to the complexity of the Iso liquid, a new kind of isotropic liquid was discovered in 2014 that is chiral, even though the molecules forming it are not.^[^ ^42^, ^43^ ^]^ The phase, labeled here Iso*, is separated from the normal nonchiral liquid (Iso) by a clear phase transition. Iso* was observed at temperatures above one of the bicontinuous phases mentioned above and often only on cooling.^[^ ^11^ ^]^ In the present compounds, the Iso–Iso* transition is detected in all but one nonpolymer as a small asymmetric DSC peak colored red in Figure 1c and marked by an asterisk. So far little is known about the nature of the Iso* phase or of its transition.^[^ ^44^ ^]^

It is fortunate that in our compounds, as in a previous report,^[^ ^5^ ^]^ both of the “unusual” isotropic liquid phases are present, the “cybotactic Iso” and the chiral Iso*. This helps highlight their difference and better understand their relationship. It should be mentioned, though, that the Iso* phase observed in most current compounds is actually metastable, with the equilibrium Cub–Iso temperature virtually coinciding with that of the Iso*–Iso transition on heating (Figure S6). However, in Vin7‐10 and in some previously reported cases,^[^ ^45^ ^]^ Iso*–Iso transition occurs ca. 10 K above the LC–Iso* transition on heating, with Iso* the thermodynamically stable phase.

### Additional Experimental Results on Current Compounds

Additional experimental results related to the *C*_p_ maximum in the present compounds are summarized in Figure 4. Temperature evolution of small‐angle X‐ray scattering is shown in Figure 4a–c for the dimer Di7‐12 and polymers Si9‐10 and Si9‐12, respectively. These cases were chosen to demonstrate the presence of pre‐assembly in melt not only above bicontinuous but also above smectic and columnar LC phases. In all cases, the scatter is seen to undergo pronounced narrowing as the Iso–LC transition temperature is approached from above. This indicates an accelerated increase in cluster size on cooling the melt. Based on the scattering curves, coherence length ξ(*T*) was calculated and compared with theory, as will be shown further below.

Figure 4d shows the temperature dependence of optical rotation angle *φ* of Vin7‐12 starting with no rotation in the Iso melt, rising sharply at the transition to the chiral Iso* melt, and then gradually level off on further cooling. This behavior provides the so far missing information on the nature of the Iso–Iso* phase transition, showing that it is clearly second‐order. Furthermore, it is interesting that upon the subsequent 1st‐order transition to the triple‐network *I*23 cubic, there is no noticeable further change in *φ*. This suggests that the local structure of the Iso* liquid closely resembles the long‐range structure of the *I*23, i.e., that it already contains sizeable fragments of isochiral networks.

The 2nd‐order nature of the Iso–Iso* transition is also indicated by temperature evolution of circular dichroism, shown in Figure 4e. The CD‐induced ellipticity of 315 nm UV light passing through a thin film of Vin7‐12 is shown both on heating and cooling. On heating, the *I*23 cubic melts directly into the achiral Iso liquid, the CD signal showing a steep yet finite‐width pre‐transitional decay and final disappearance. On cooling, CD develops more slowly below *T*_Iso–Iso*_, again consistent with the critical nature of the Iso–Iso* transition. As with optical rotation, no jump in CD is observed at *T*_Iso*–_ *_I_*_23_. One should bear in mind that the Vin7‐12, like all other current compounds, is achiral, and that in repeated experiments the sign of CD changes randomly.

Near‐equilibrium calorimetry was performed in the *T‐*range of interest by modulated DSC (also known as AC‐calorimetry), where, superimposed on a very slow linear cool (0.04 K min^−1^), was a sinusoidal oscillating temperature deviation of 0.07 K amplitude. As an example, two MDSC thermograms of Di3‐10 are shown in Figure 4f, one with the oscillation period 20 s and the other with 60 s. The top traces show the part of the heat capacity based on heat flow that reverses in each cycle (*C*_p_^rev^). The bottom traces show the small phase lag between temperature and heat flow. While with a 20 s period, there is still a residual phase lag around the *C*_p_ hump and the Iso–Iso* transition, when the period was extended to 60 s, the phase signal remained at zero, meaning that the system was kept at equilibrium with all relaxation times below 1 min. Incidentally, because of the hysteresis of the 1st‐order Iso*–*I*23 phase transition exceeding the oscillation amplitude, the heat flow from released transition enthalpy is nonreversing, hence not showing either in *C*_p_^rev^ or in the phase, leaving only the step drop in *C*_p_^rev^ at the transition at 75 °C. The *C*_p_^rev^ curves from MDSC are used in theoretical analysis, as described below.

Although against the odds, we also tried to see if it might be possible to image the Iso* liquid. We have polymerized a monomer compound (Vin9‐7F) by UV irradiation in the Iso* state and then quenched it to room temperature. We used freeze‐fracture transmission electron microscopy and atomic force microscopy. However, neither method gave any recognizable texture, as can be seen from the images in Figures S38 and S39.

### Theory

**Figure 5:**
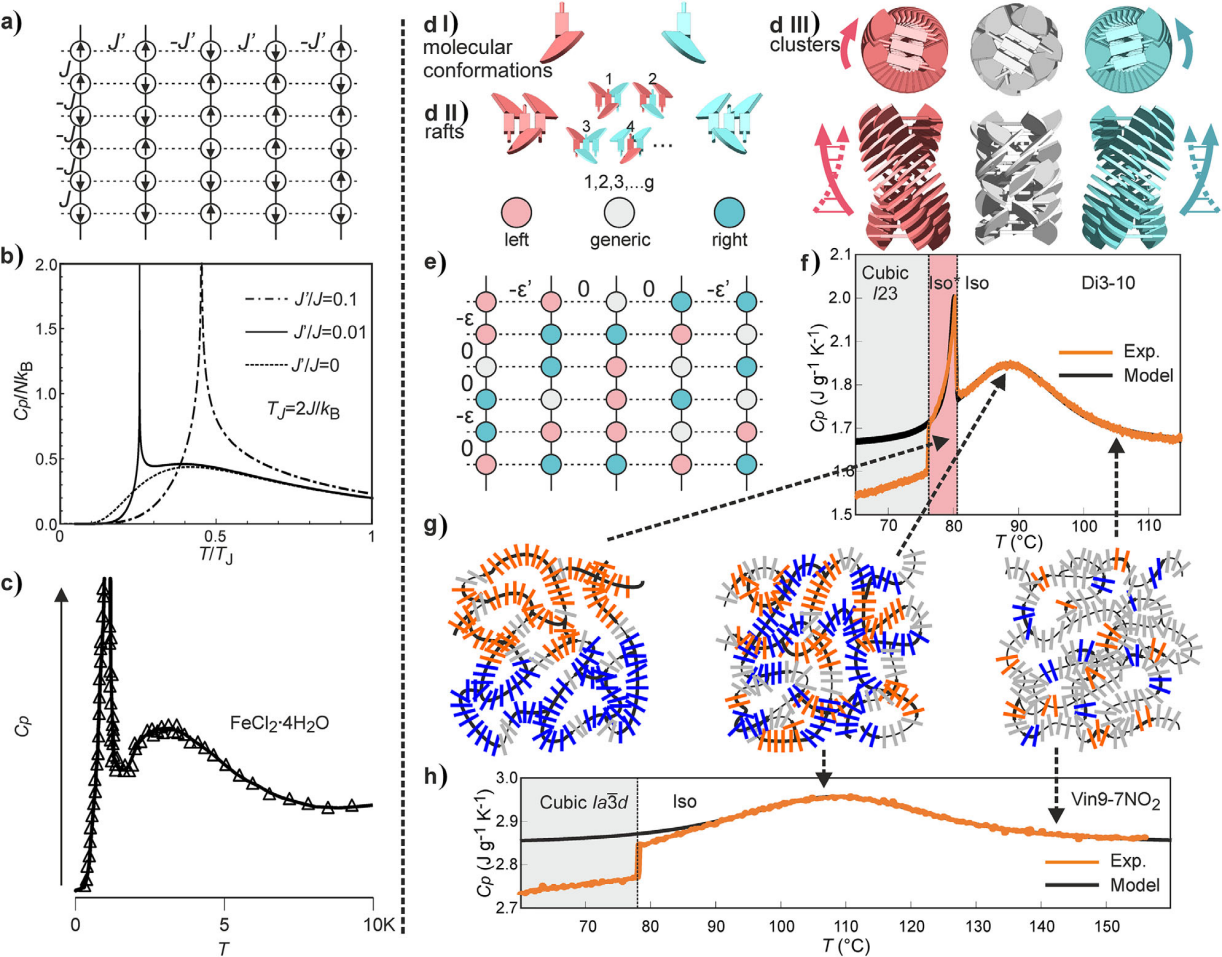
a) The original 2D Ising model,^[^ ^47^ ^]^ where each lattice point carries a spin with nearest neighbor interactions stronger vertically (*J*) than horizontally (*J’*). b) *C*_p_ versus *T* for different *J’*/*J* ratios. Broken line: *J’*/*J *= 0 (1D Ising). Solid: *J’*/*J *= 0.01 (both *C*_p_ hump and order–disorder transition present). Dot‐dash: *J’*/*J *= 0.1 (broad *C*_p_ maximum disappears). c) The experimentally observed order–disorder transition below the Schottky anomaly in FeCl_2_·4H_2_O due to spin ordering.^[^ ^48^ ^]^ d)(I) Two antichiral conformations of Vin*m‐n* (schematic, as in Figure 1b). d)(II) Left and right: two antichiral rafts; middle: example of four out of *g* generic rafts. Henceforth, generic rafts are colored grey. d)(III) Segments of twisted columns of stacked rafts that can be purely left‐ or right‐handed, or any state in between; top: top view, bottom: side view. e) Modified lattice model of Iso and Iso* phases, where each lattice point is a molecular raft that is either left‐handed (red), right‐handed (blue), or generic (white). The interaction between two lattice sites of the same hand reduces system energy, with the interaction energy in the vertical direction (or along the column) ε much stronger than that in the horizontal direction (or between neighboring columns) ε′. f) and h) Comparison between theoretical and experimental temperature‐dependent heat capacity (*C*_p_^rev^) for Di3‐10 and Vin9‐7NO_2_, respectively. The *T*‐scales are matched for easy comparison. g) Schematic depiction of self‐assembly of the network phases in achiral and chiral melt. Rafts are represented by short, thick lines (colors as in (d and e)). The rafts pack into quasi‐1D columns (long, curvy black lines) and form a 3D aperiodic network. At higher temperatures (right), the rafts are mostly generic, i.e., of mixed chirality. At the same time, chiral enantiopure rafts have very little correlation between them. On cooling (middle), chiral rafts become more correlated and aggregates grow. Further cooling (left) results in the Iso* phase. Dashed arrows point to regions on the MDSC curves.

An example of a thermodynamic system that could display both a broad *C*_p_ hump and a sharp order–disorder (OD) transition was, in fact, given by Onsager in his seminar paper on 2D Ising model.^[^ ^47^ ^]^ The model considered only nearest neighbor spin interactions on a rectangular lattice, with interaction energy different in vertical (*J*) and horizontal (*J*’) directions (Figure 5a). By increasing *J’*/*J*, thermodynamic behavior of the system changes from the 1D (*J’*/*J *= 0), via anisotropic 2D (0 < *J’*/*J *< 1) to isotropic 2D (*J’*/*J *= 1). In 1D, there is no OD transition, and a continuous *C*_p_ curve with a maximum at *T* = ∼0.83 *J k*_B_^−1^ is predicted (Figure 5b, dotted line). The diffuse part is normally known as “Schottky anomaly,” and in the simplest case demonstrated by a 1D Ising model with only nearest neighbor spin interactions. Through the broad *C*_p_ maximum, correlation length increases with decreasing temperature. An OD transition is found as soon as a nonzero *J*’ is introduced. If *J*’ is kept sufficiently small, i.e., ≤0.01 *J*, the OD transition is clearly separated from the original *C*_p_ maximum, appearing at a lower *T* (Figure 5b, solid line). At higher *J’*/*J* ratios, the broad hump disappears (dot‐dash line). Such behavior has indeed been observed in magnetic systems with anisotropic spin interactions at temperatures near 0 K (Figure 5c).^[^ ^48^ ^]^

A qualitative analogy can be drawn between Onsager's model and self‐assembly of rod‐like molecules, between which there is strong anisotropy in interaction. The formation of columnar clusters through strong core–core interaction can be treated as pseudo‐1D, so that only a broad *C*_p_ hump is expected. The correlation length in such clusters increases continuously on cooling until it reaches a critical value where the much weaker lateral interactions between clusters accumulate sufficiently to trigger the transition to an LRO state.

However, there are quantitative difficulties using the Onsager model directly. The *C*_p_ hump in 1D Ising is very broad, its width (FWHM) is even larger than the temperature of its maximum, *T*_max_. By contrast, our experimental FWHM is typically <10% of *T*_max_. Furthermore, in Onsager's model, the transition to ordered phase occurs at a temperature much lower than *T*_max_.

Although our molecules adopt many conformations in the Iso and the LRO phases, the base for our modification of the Onsager model is the fact that these conformations can be divided into two groups, either left‐ or right‐handed (blue and red in Figure 5d(I)). The key barrier separating the two is the clash between atoms circled in Figure 1a. Two to four of such molecules, packing parallel to each other, form a raft that can be left‐ or right‐handed (Figure 2a and Figure 5d(II)). The rafts, successively rotated by ∼10°, stack to form chiral columnar segments (Figure 5d(III)).^[^ ^15^, ^16^ ^]^

We start our model by describing self‐assembly of rod‐like molecules into columnar nanoclusters in the melt as ordering of molecular rafts on a 1D lattice. In “chiral” rafts, all molecules are in either left or right conformation, while “generic” rafts contain a mixture (Figure 5d(II)). These are symbolized by red, blue, and white circles in Figure 5e. We also assume that the generic state has degeneracy *g *> 1, i.e., it has *g* thermodynamically equivalent configurations. For simplicity, we assume that in isolation, the energy of all three raft states equals 0 and is lowered by ε  = *k*_B_ *T*_ε_ only when two isochiral rafts are neighbors. The partition matrix for a pair of neighboring units can be written as:

(1) $$V=\left(\begin{array}{ccc} e^{\beta \varepsilon } & 1 & g\\ 1 & e^{\beta \varepsilon } & g\\ g & g & g^{2} \end{array}\right)\;$$

here, $\beta =\frac{1}{k_{B}T}$. The partition function for a system of *N* units is then

(2) $$Z_{N}=Tr\left(V^{N}\right)$$

*Z*_N_ is dominated by the largest eigenvalue of matrix *V* which is $\frac{\lambda +1+g^{2}+\sqrt{{\left(\lambda +1-g^{2}\right)}^{2}+8g^{2}}}{2}$ and

(3) $$\ln Z_{N}=N\ln\frac{\lambda +1+g^{2}+\sqrt{{\left(\lambda +1-g^{2}\right)}^{2}+8g^{2}}}{2}$$

here, λ  = *e* ^βε^. Based on the partition function, equations for system's energy *E* and heat capacity *C* can be derived; for details, see Section 8 (Supporting Information). The results match well the experimental *C*_p_(*T*) for all compounds, revealing interesting information on the effect of chemical structure on the self‐assembly process.

Figure 5h shows an example for Vin9‐7NO_2_, which does not display the Iso–Iso* transition. The experimental curve is virtually at equilibrium, derived by modulated DSC. The fit is almost perfect at *T *> 90 °C, the deviation below being due to transition to the $Ia\bar{3}d$ cubic phase at 78.1 °C and associated pre‐transitional effects. Fitting parameters are ε = 14.6 kJ mol^−1^, *g *= 9.75, and mass of the unit (raft) *M*_raft_ = 2850 g mol^−1^. While ε and *g* values determine *T*_max_ and the FWHM of the *C*_p_ hump, respectively, *M*_raft_ is linked to its height. As the mass of Vin9‐7NO_2_ molecule is 926 g mol^−1^, each raft in the melt should contain ∼3 molecules. This fits well with the above network model of bicontinuous phases (Figures 2a and 4d).^[^ ^9^, ^11^, ^15^, ^49^ ^]^ Considering the energy of typical van der Waals bonds augmented by π–π interaction between aromatic groups, ε= 14.6 kJ mol^−1^ between two rafts is of the expected order of magnitude. The degeneracy *g* of ∼10 fits roughly the number of molecules in the raft too. Assuming that each molecule can adopt either a left‐ or a right‐handed conformation, then out of the total 2^3 ^= 8 states for a 3‐molecule raft, there would be 6 mixed (generic) states for any one, say, left‐handed state.

Now we consider the weaker interactions between rafts in neighboring columns that trigger the critical Iso–Iso* transition. Physically in our compounds, these could be attributed primarily, but not exclusively, to weak van der Waals interactions between chain ends. There is a critical value *p*_c_ of the probability (*p*) of a raft adopting the chiral state above which the LRO will set in, resulting in a further reduction in system energy. The energy drop, when the system is fully ordered (*p*  =  1), is the interaction energy ε′ between two isochiral rafts in neighboring columns. While solving precisely our model in Figure 5e is beyond the scope of this paper, we can speculate on the effects of ε′ on ordering of the Iso* phase by assuming that the reduction of system's energy (Δε) is a function of probability *p* according to a power law:

(4) $$\Delta \varepsilon ={\left(\frac{p-p_{c}}{1-p_{c}}\right)}^{\delta }\varepsilon^{\prime },\;p_{c}<p<1$$

This energy change can be affected by changing the value of λ from *e* ^βε^ to *e* ^β(ε + Δε)^. Through iteration, we can arrive at a set of self‐consistent *p*, λ, *E*, and *C* values at different temperatures numerically (for details, see Equations S9–S13 in Supporting Information). The results are able to explain quantitatively both the *C*_p_ hump in Iso as well as the 2nd‐order transition to Iso*. An example is given in Figure 5f for Di3‐10 (see also Figures S10–S14).

In our system, the Iso* phase, when it is observed, is always below the *C*_p_ hump, due to the fact that *ε’*/*ε* is small. For completeness, in Figure S8 we show three *C*_p_ curves calculated by our theory for *ε’*/*ε* = 0, 0.0343, and 0.1. As in Figure 5a, with no weak interactions, only a broad hump is seen. As *ε’*/*ε* = 0.0343 falls within the range of our current monomers and dimers, the 0.0343 curve is of the same type as observed in those compounds (Figure 1). However, when *ε’*/*ε* = 0.1, the hump is lost under the substantially enhanced transition peak.

### Interpretation of Experimental Results

The best‐fit parameters to experimental MDSC curves (Figures 4 and S10–S14) are listed in Table 1. The strong interaction energies ε have similar values (∼14 kJ mol^−1^) per monomer repeat unit (MRU) for Vin molecules (1 MRU) and Si polymers and somewhat higher (∼17 kJ mol^−1^) for dimers (i.e., ∼34 per molecule). This is plausible since, according to the best‐fit *M*_raft_, in the dimers there are ∼4 MRUs in each raft (2 molecules) instead of ∼2–3 for Vin molecules and polymers. The increased ϵ; therefore, comes from the increased lateral interaction energy within the raft. This also makes the degeneracy *g* of the generic state in dimers the highest (15–20). This explains the very obvious narrowing of the *C*_p_ hump in the dimers (Figure 1c). In contrast, *g* is the lowest, *g *= 7.1, in polymer Si9‐12, consistent with its small *N*_mono_ and, being a polymer, its restricted number of conformations (hence *g*) in the melt. As *C*_p_ maximum happens at *T*_max_≅ε/(2*k*_B_ln *g*), for Si9‐10 and Si9‐12, it is shifted to much higher temperatures compared to the nonpolymers. For monomers and dimers, *T*_max_ values are similar since an increase in ε (*T*_max_ ↑) is compensated by an increase in *g* (*T*_max_ ↓), both determined by *N*_mono_. Interestingly, replacing a hydrogen with fluorine in Vin9‐7F or with NO_2_ in Vin9‐7NO_2_, reduces *g* in both cases, attributed to dipolar coupling restricting the number of configurations in the melt. As in the conformationally restricted polymers, the small *g* value broadens the *C*_p_ hump, meaning less cooperativity of the assembly process. The strong polarity and electron‐withdrawing effect of NO_2_ (high Hammett constant^[^ ^50^ ^]^) seem to increase not only ε of the rafts in melt but also in the LC, lifting the Iso–LC temperature above *T*_Iso–Iso*_.

**Table 1:** Comparison of best‐fit parameters for different compounds.^a)^

Compound	*M*_mono_ (g mol^−1^)	*M*_raft_ (g mol^−1^)	*N*_mono_	*T*_max_ (°C)	ε (kJ mol^−1^)	*g*	*T*_Iso–Iso_^*^ (°C)	ε′ (J mol^−1^)	*p*_c_	δ
Vin7‐12	1060	2850	2.7	85.7	14.4	11.0	70.6	499	0.87	1.20
Vin9‐7F	895	1300	1.4	80.0	12.0	7.42	63.1	291	0.83	0.94
Vin9‐7NO_2_	922	2850	3.1	108.7	14.6	9.75	–	–	–	–
Vin9‐9	961	1950	2.2	87.0	13.9	10.0	77.4	274	0.78	0.95
Di3‐10	987	3750	3.8	88.6	16.5	15.5	80.6	407	0.83	1.25
Di7‐12	1127	5100	4.5	85.4	17.9	20.0	78.9	457	0.84	1.60
Si9‐12	1000	2400	2.4	163.1	14.5	7.1	–	–	–	–

^a)^ *M*_mono_ = mass of monomer repeat unit (1 MRU = 1 Vin = 1/2 Di molecule), *M*_raft_ = mass of raft, *N*_mono_ = number of MRUs in raft, *T*_max_ = temperature of *C*_p_ maximum, ε and $\varepsilon^{\prime }$= interaction energies between rafts in same and neighboring columns, respectively, *g* = degeneracy of generic state, *p*_c_ = critical probability of raft being in correct chiral state, *δ* = exponent in Equation (4).

ε′ has similar values for different compounds, and so does *T*_Iso–Iso*_. This is understandable since all studied compounds contain the same mesogen. *T*_Iso–Iso*_ follows the same trend as *T*_max_, i.e., both are higher when the mesogen is longer and more rigid.^[^ ^11^, ^45^ ^]^

The self‐assembly sequence on cooling of our compounds in the melt is schematically presented in Figure 5g. At higher temperatures in the melt, assuming the rafts have already formed, they are mostly generic, while chiral rafts are very weakly correlated. On cooling, chiral rafts become more correlated and start to aggregate in larger clusters. At the *C*_p_ maximum, more than half of the rafts are in chiral states, and their average cluster length is ∼10 ± 2 rafts (∼4.5 ± 1 nm), without any long‐range segregation of left‐ and right‐handed segments. However, on further cooling, such separation does happen due to the weaker ε′ inter‐columnar interactions, resulting in the Iso–Iso* transition. At *T*_Iso–Iso*_, more than *p*_c _= 80% of the rafts are chiral, and the average cluster length is ∼20 ± 5 rafts (∼9 ± 2 nm).

**Figure 6:**
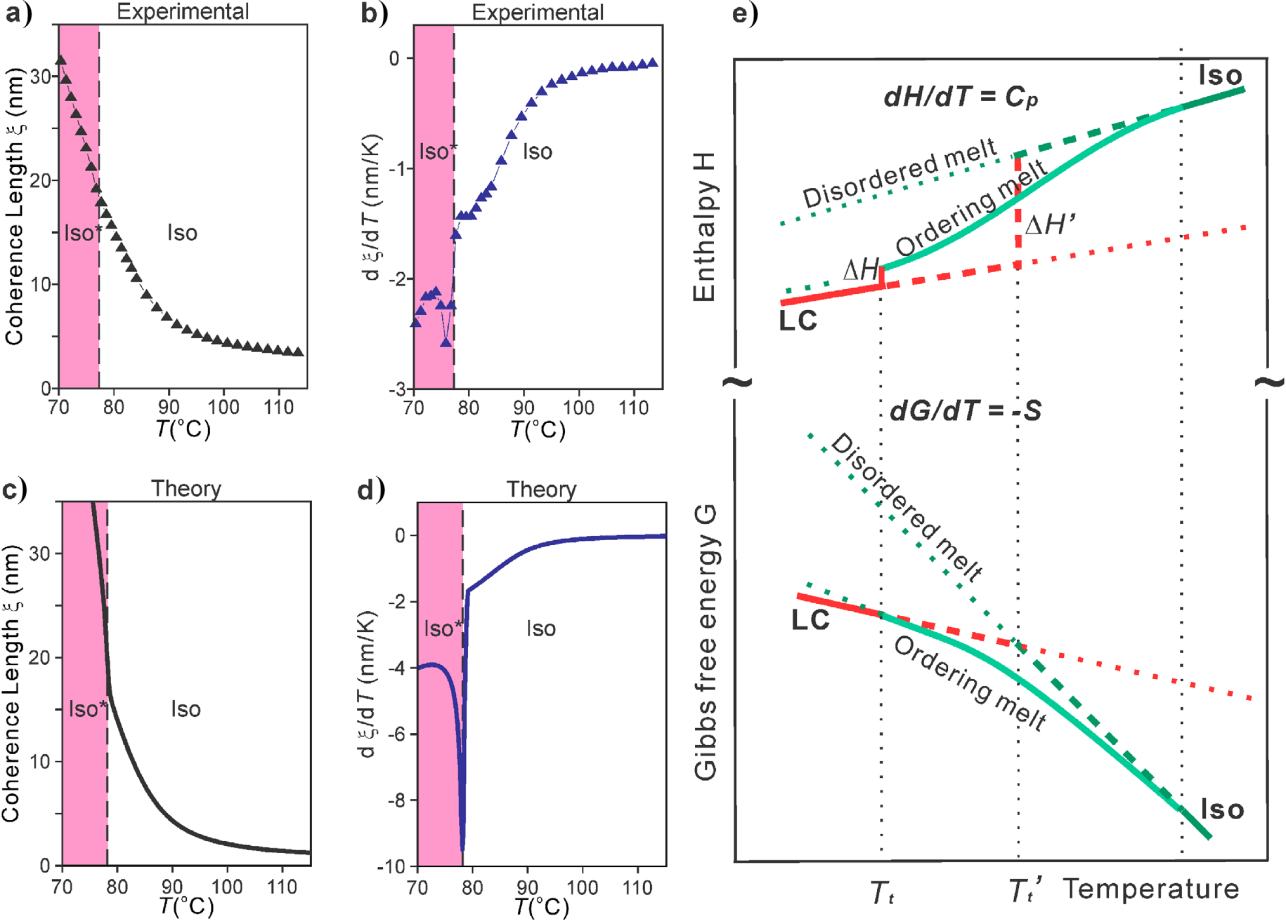
a) and c) Temperature evolution of coherence length ξ (a) from SAXS (compound Di7‐12) and (c) from theory. b) and d) Derivative *d*ξ/*dT* from (b) experiment and (d) theory. ξ increases on cooling, with a stepwise change in slope *d*ξ/*dT* at the Iso–Iso* transition. A small step at *T*_Iso–Iso*_ in the actual experimental ξ(*T*) curve can also be noticed in the close‐up of the ξ(*T*) curve shown in Figure S5 (right). e) Schematic *T*‐dependence of free energy (bottom) and enthalpy (top) for a case of a conventional 1st‐order structural phase transition (straight lines crossing at *T*_t_ *’*), and a case of continuous self‐assembly in the melt displaying a *C*_p_ hump and a separate transition to the ordered state at *T*_t_ with a significantly reduced transition enthalpy Δ*H* (curved line). The path in the former case is marked by dashed lines and in the latter by full lines. Dotted lines are extensions into unstable range.

Based on the profound narrowing of the diffuse SAXS as the compounds are cooled through the *C*_p_ hump (Figure 4a), coherence length (“crystal size”) *ξ* was obtained using the diffuse peak's width and the Scherrer equation. Experimental *ξ*(*T*) is plotted in Figure 6a for Di7‐12, along with its derivative in Figure 6b. For comparison, shown in Figure 6c is *ξ* versus *T* calculated from our model. The match between theory and experiment is very satisfactory. Noteworthy in particular is the small‐sharp negative peak in *dξ*/*dT*, seen in both experimental and theoretical curves, signaling a discontinuous step‐up in *ξ* with decreasing *T*, coinciding with the Iso–Iso* transition. Also notably, for all studied compounds, at Iso–Iso* transition *ξ* has virtually the same value of ∼15 nm, similar to that calculated from the theory.

Comparison of heating and cooling DSC traces for another Vin monomer, Vin7‐10 (Figure S6), shows reversibility of the *C*_p_ hump and the Iso* transition, as well as the hysteresis of the 1st‐order LC transition. The shape and reversibility of the Iso–Iso* transition are consistent with its 2nd‐order nature. This is confirmed by MDSC, where full reversibility of *C*_p_(*T*), indicated by zero heat‐flow phase lag, is observed on a 60 s time scale (Figure 4f). Moreover, 2nd order character of the Iso–Iso* transition is confirmed chirooptically, both by optical rotation angle *φ*(*T*) within a domain (Figures 4d and S7) and by CD spectroscopy (Figure 4e).

It is worth noting that *T*_Iso–Iso*_ is found within a relatively narrow range of (74 ± 6) °C, irrespective of whether the compounds are monomers or dimers and, seemingly, uncorrelated with the *T*_Iso–LC_. Thus, in compound Vin9‐7NO_2_ and in the polymers, the Iso–Iso* transition is not observed even on cooling as *T*_Iso–LC_ is raised above *T*_Iso–Iso*_. Why *T*_Iso–LC_ is raised is clear from *T*_Iso–LC_ = Δ*H*/Δ*S*, where Δ*H and* Δ*S* are enthalpy and entropy of the transition. As already mentioned, in Vin9‐7NO_2_ the strong dipolar interaction between nitro groups lowers *H*_LC_. In the polymers, it is *S*_Iso_ that is lowered relative to that of the monomers and dimers. The small variation in *T*_Iso–Iso*_ suggests that it is dominated by the nature of the rod‐like mesogenic group, which is what all our compounds have in common.

### Why Hump?

Although the answer to this question is implicit in the theory described above, here we try to give a qualitative answer, a simple physical picture of the phenomenon. A *C*_p_ maximum means that heat capacity decreases both on heating and on cooling. On heating, the decrease at high *T* happens because all clusters are already disassembled and there is no further de‐aggregation left to absorb energy. Of course, the baseline *C*_p_ still continues to increase as new degrees of freedom at higher energy become accessible. But why the drop in *C*_p_ at lower *T*? Because although the size of the clusters and the coherence length continue increasing on cooling below *T*_max_, this increase happens mainly through merger of already existing clusters, not requiring formation of many new bonds. Or, coming from below, upon heating toward *T*_max_ *C*_p_ increases because an ever‐increasing number of bonds must be broken to keep reducing the cluster size and maintain equilibrium. This feature is what distinguishes the phenomenon of a *C*_p_ maximum from the usual pre‐transitional ordering, where *C*_p_ only increases as the transition *T* is approached from above. The drop in *C*_p_ below *T*_max_ will be greater the more suppressed the temperature of the transition to LRO.

In Figure 6e, a simple schematic thermodynamic scheme illustrates the difference between an ordinary 1st‐order phase transition, such as melting/crystallization, where free energy versus *T* of the melt and the crystal can be approximated by straight lines (strictly they slowly curve), where the melting enthalpy changes by a large amount Δ*H’* at the intersection of the two lines at *T*_t_ *’*. In contrast, cluster formation stabilizes the melt, lowering its enthalpy and relative free energy as it is cooled through the *C*_p_ maximum. This delays its intersection with the free energy of the ordered LC. As a consequence, the enthalpy Δ*H* of the transition that eventually happens at a lower temperature *T*_t_ is significantly reduced.

Finally, while in Section ‘*C*_p_ Hump in Other Systems,’ we mentioned other examples of systems where formation of a LRO phase is preceded by a *C*_p_ hump, there is a particularly interesting group of mesogens whose complex LC phases form from the melt without an obvious *C*_p_ anomaly. These are bent‐core (BC) mesogens forming smectic phases that can show spontaneous chirality and polarity. There is some frustration in these phases, but in contrast to the straight‐core molecules whose interactions favor flat layers, in BC compounds they favor twisted or saddle‐deformed layers in the ground state. Their bend^[^ ^51^ ^]^ or saddle‐splay^[^ ^52^ ^]^ elastic constant is negative. In some BC compounds, this results in layer stacks that are twisted or have Gaussian curvature with no true LRO, respectively, in the so‐called helical nanofilament (HNF)^[^ ^53^ ^]^ and dark conglomerate (DC)^[^ ^54^ ^]^ phases. Notably, however, unlike in, e.g., a TGB phase of straight‐rod but chiral molecules, in BC compounds it is the flat‐layer phase, such as SmCP (B2), that forms directly from Iso melt and is stable at high *T*, where the orientational freedom that flat layers allow dominates. Only at lower *T*, the energy‐favored layer distortion becomes dominant, leading to HNF or DC phases through quite a spectacular sequence of structural jumps accompanied by a series of unusually sharp exotherms.^[^ ^55^ ^]^ The *C*_p_ hump in the melt is absent, since this inverse frustration plays out below rather than above the *T*‐range of the LRO phase.

## Conclusions

Using a series of newly synthesized LC‐forming monomers, dimers, and polymers, we have shown the consistent presence of a pre‐assembly *C*_p_ maximum in Iso melt and arrived at a satisfactory quantitative description of the associated self‐assembly process on molecular level. Also, the transition to the recently discovered chiral Iso* liquid, shown here to be second order, fits naturally into the proposed theoretical framework. In addition to the noted thermodynamic analogy between the current systems and those in TGB LCs, blue phase LCs, and magnetic crystals, our experiments show that pre‐assembly of a similar kind, exhibiting a previously unreported *C*_p_ anomaly in melt, can also be seen in systems preceding the formation of other phases: columnar LCs, some smectic LCs, as well as some other phases still to be reported. The theory developed here can be easily adapted to reproduce quantitatively the many similar experimental observations in condensed matter. This work also illustrates the wide applicability of Onsager's idea that systems with highly anisotropic interactions could have their ordering split into two stages. In fact, from the diversity of systems showing the *C*_p_ anomaly, we see that anisotropy is not a necessary prerequisite, as long as strong and much weaker interactions coexist. When the strong interactions cannot lead to LRO by themselves because the local clusters formed are either 1D or frustrated in their 3D packing, the establishment of LRO can only be triggered at a lower temperature. That happens when correlation length of clusters increases sufficiently for the combined force of the weak interactions to tip the balance in favor of LRO.

## Conflict of Interests

The authors declare no conflict of interest.

## Supporting information



Supporting Information

## Data Availability

The data that support the findings of this study are openly available in figshare.com at https://doi.org/10.6084/m9.figshare.26086531.v1.
