# Supplementary material for: On the Mechanism of Soft Self‐Assembly from Melt: The Ubiquitous Heat Capacity Hump and Spontaneous Melt Chirality
Source: Angew Chem Int Ed Engl. 2025 May 15;64(28):e202505548. doi: 10.1002/anie.202505548 (PMC12232891; doi:10.1002/anie.202505548)
Supplement: Supplementary file 1 — Supporting Information [file ANIE-64-e202505548-s001.docx]

**Supporting Information**

**On the Mechanism of Soft Self-assembly from Melt: The ubiquitous Heat Capacity Hump and Spontaneous Melt Chirality**

Yi-nan Xue^1^, Xiang-bing Zeng^2^*, Bo-wen Wu^1^, Ya-xin Li^3^, Liliana Cseh^4^, Shu-gui Yang^1^, Jie Liu^1^, Gillian A. Gehring^5^, Feng Liu^1^, Goran Ungar^1,2^*

^1^ Shaanxi International Research Center for Soft Matter, State Key Laboratory for Mechanical Behaviour of Materials , Xi'an Jiaotong University, Xi’an 710049, China

^2^ School of Chemical, Materials and Biological Engineering, University of Sheffield, Sheffield S1 3JD, U.K.

^3^ School of Chemistry and Chemical Engineering, Henan University of Technology, Zhengzhou 450001, China

^4^ Romanian Academy, Coriolan Dragulescu Institute of Chemistry, Timisoara 300223, Romania

^5^ School of Mathematical and Physical Sciences, University of Sheffield,, Sheffield S3 7RH, U.K.

Correspondence to: [g.ungar@sheffield.ac.uk](mailto:g.ungar@sheffield.ac.uk), [g.ungar@xjtu.edu.cn](mailto:g.ungar@xjtu.edu.cn), [x.zeng@sheffield.ac.uk](mailto:x.zeng@sheffield.ac.uk)

Contents

[1. Table S1. Isotropization enthalpies of typical nematics 2](#_Toc196986773)

[2. Additional polarized optical micrographs 2](#_Toc196986774)

[3. Additional X-ray diffraction data 4](#_Toc196986775)

[4. Additional calorimetric data 10](#_Toc196986776)

[5. Additional chiroptical data 12](#_Toc196986777)

[6. Additional theory 12](#_Toc196986778)

[7. Additional comparison of theory and experiments 15](#_Toc196986779)

[8. Methods of physical and structural characterization 17](#_Toc196986780)

[9. Synthesis and analytical data 18](#_Toc196986781)

[10. Additional imaging 54](#_Toc196986782)

[11. References 55](#_Toc196986783)

1. Table S1. Isotropization enthalpies of typical nematics

**Table S1.** Examples of typical enthalpies of nematic-isotropic tansitions from literature – compare with LC-Iso transitions in Fig. 1 of main text

| Compound | ΔH | Reference |
| --- | --- | --- |
|  | (J/g) |  |
| CBO11O.Py | 0.73 | S^[[1]](#endnote-1)^ |
| CBO11O.Py | 2.62 | S^[[2]](#endnote-2)^ |
| CBO9O.Py | 1.82 | S2 |
| CBO7O.Py | 1.58 | S2 |
| PAA | 2.84 | S^[[3]](#endnote-3)^ |
| AAD | 2.47 | S3 |
| MBPA | 1.71 | S3 |
|  |  |  |
| average | **1.97** |  |
|  |  | PAA = p-azoxyanisole |
|  |  | AAD = anisaldazine |
|  |  | MBPA = N-p-methoxybenzylidene-p-phenylazoaniline |

1. Additional polarized optical micrographs


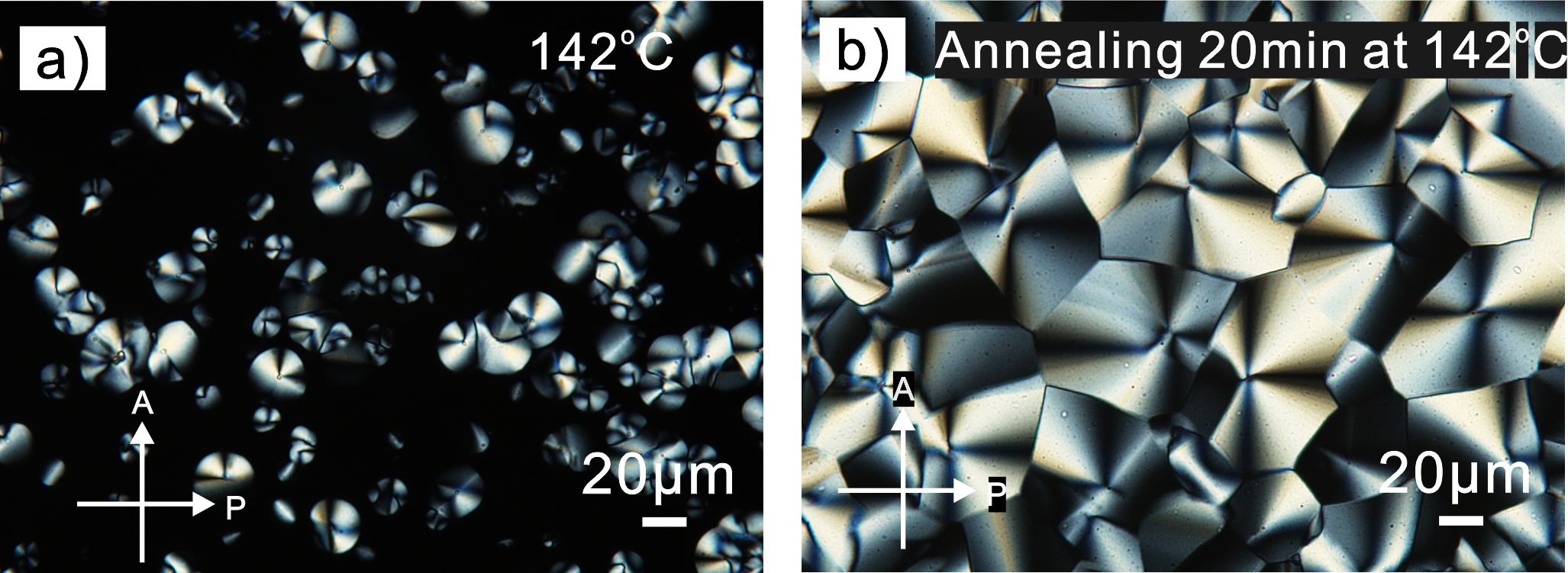


**Figure S1.** Textures of the Col_hex_ phase of Si9-12 between crossed polarizers at 142 ^o^C, **a.** the incipient stage of texture; **b.** after annealing 20 min at 142 ^o^C.


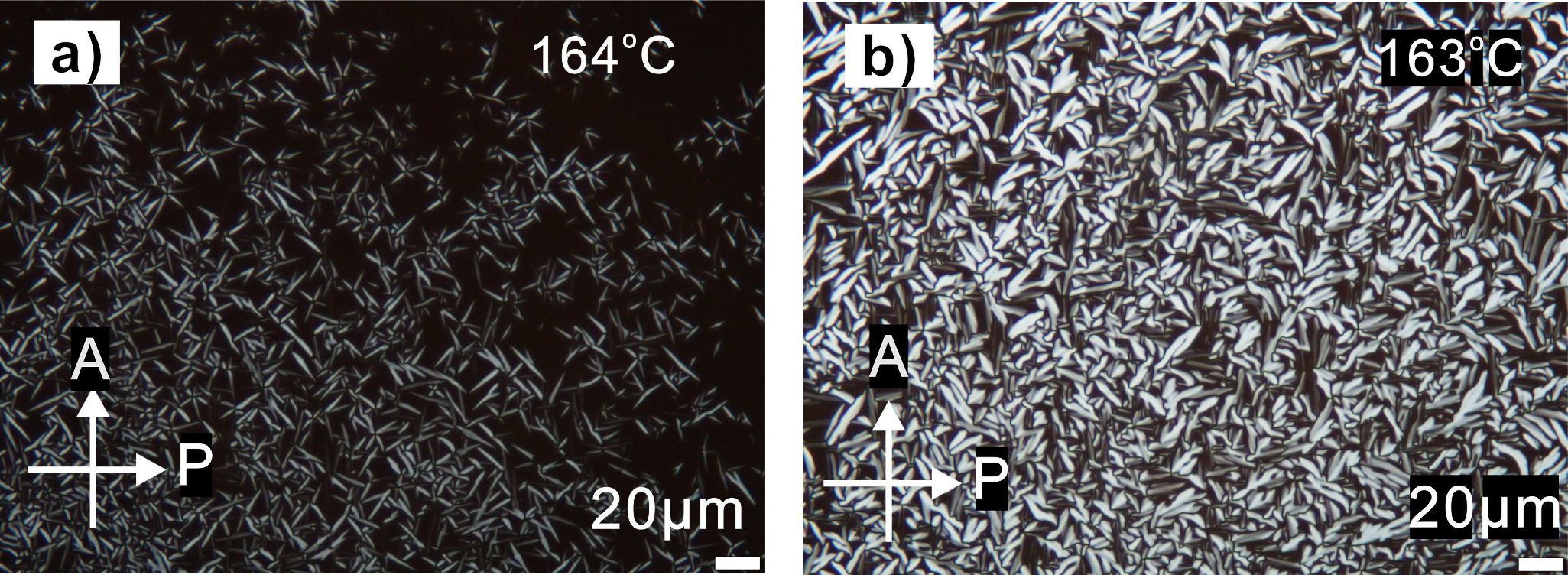


**Figure S2.** Textures of the smectic phase of Si9-10 between crossed polarizers: **a.** batonnets at the incipient stage of Sm phase growth at 164 ^o^C through screw dislocation; **b.** at 163 ^o^C.

1. Additional X-ray diffraction data


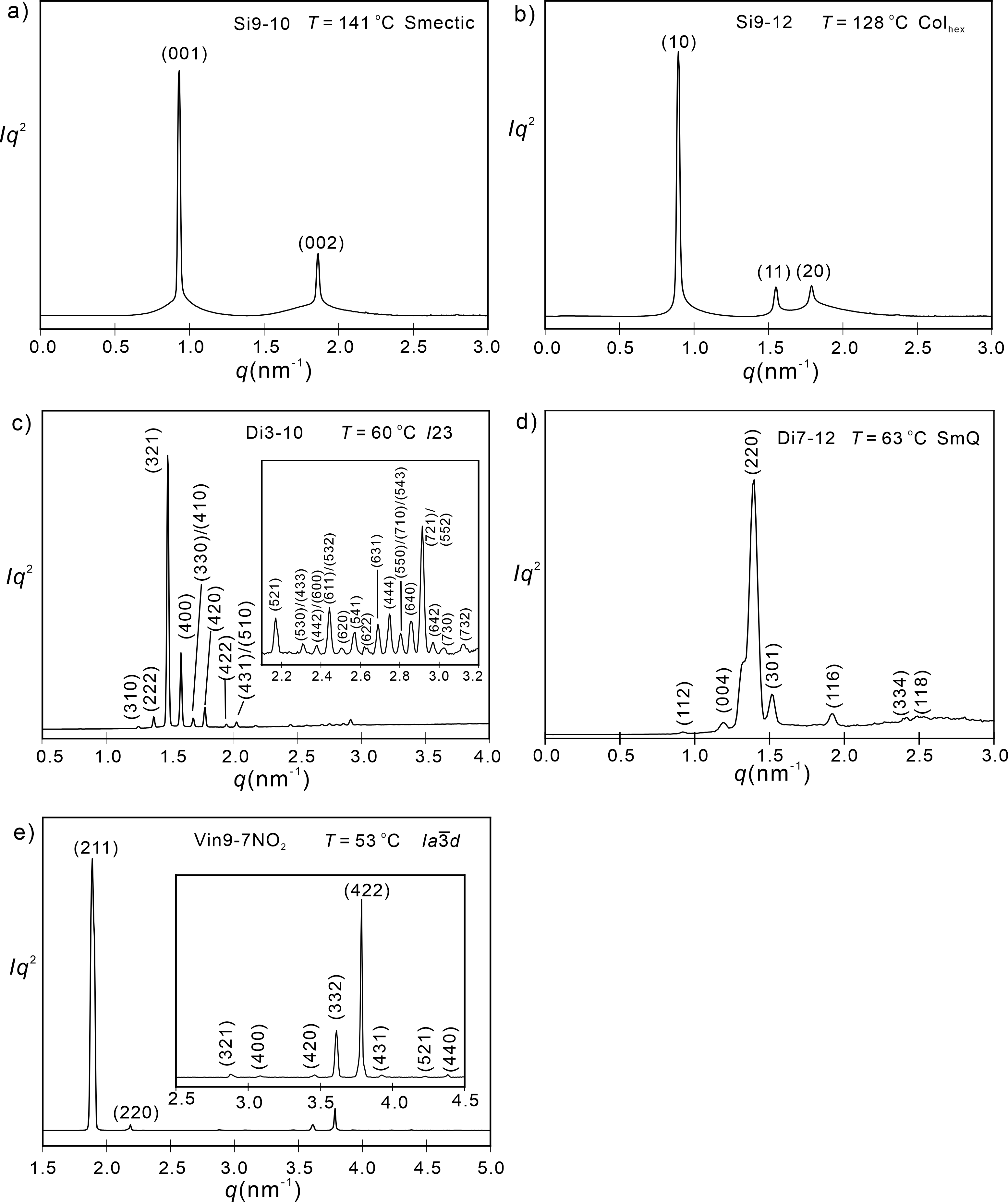


**Figure S3.** Powder SAXS diffractograms of **a.** polymer Si9-10 at 141^o^C, Smectic phase; **b.** polymer Si9-12 at 128 ^o^C, Col_hex_ phase; **c.** compound Di3-10 at 60 ^o^C, *I*23 cubic phase; **d.** Di7-12 at 63 ^o^C, SmQ tetragonal phase; **e.** Vin9-7NO_2_ at 53 ^o^C, $Ia\bar{3}d$ gyroid cubic phase.





**Figure S4**. Examples of fitting the experimental SAXS curves *Iq*^-1^ *vs.* *q* with a Lorentzian function for Di7-12 at four different temperatures, one below *T_Iso-Iso_** and three between *T_Iso-Iso_** and *T_Iso*-LC_.*

In order to obtain reliable values of coherence length in the melt from SAXS curves objectively, without influence of human subjectivity, for measurement of peak width, each curve was fitted automatically by Origin, using Lorentzian lineshape. To compensate for the asymmetry of the SAXS peak, the experimental peaks were divided by *q*. The resulting *Iq*^-1^ vs. *q* functions gave a very good fit to the Lorentzian, as shownin in Figure S4 by examples for compound Di7-12 at four different temperatures in the key range above and below the Iso-Iso* transition. The FWHM values were then converted back to the values corresponding to the appropriate *Iq*^2^ function. The coherence length was calculated as ξ = 2*π*/FWHM, and shown against *T* for Di7-12 in Figure S5.

**

**

**Figure S5**. Coherence length *vs.* temperature for Di7-12 obtained from SAXS curves using the fitting procedure described above. Left: full *ξ*(*T*) curve, right: detail around the Iso-Iso* transition. The straight dotted lines are to guide the eye.

**Table S2.** Cell parameters of phases for the investigated compounds.

| Compd. | Phase | *T*/^o^C | *a*/nm | *c*/nm |
| --- | --- | --- | --- | --- |
| Vin7-12 | *I*23 | 61 | 16.67 |  |
| Di3-10 | *I*23 | 60 | 15.85 |  |
| Di3-12 | SmQ | 39 | 12.09 | 20.16 |
| Di7-12 | SmQ | 63 | 12.71 | 21.06 |
| Vin9-7NO_2_ | $Ia\bar{3}d$ | 53 | 8.15 |  |
| Si9-10 | Sm | 141 | 6.76 |  |
| Si9-12 | Col_hex_ | 128 | 8.10 |  |

**Table S3.** Miller indices *(hkl)* and *d*-spacing of the diffraction peaks for the Cub_bi_/*I*23 phase observed at 61ºC in compound Vin7-12.

| Vin7-12 (*hkl*) | *d*_obs._ - spacing/nm | *d*_calc._ - spacing/nm | *d*_obs._ - *d*_calc._ |
| --- | --- | --- | --- |
| (310) | 5.27 | 5.27 | 0.00 |
| (222) | 4.82 | 4.81 | 0.01 |
| (321) | 4.46 | 4.46 | 0.00 |
| (400) | 4.17 | 4.17 | 0.00 |
| (330) | 3.93 | 3.93 | 0.00 |
| (420) | 3.73 | 3.73 | 0.00 |
| (422) | 3.41 | 3.40 | 0.01 |
| (431)/(510) | 3.27 | 3.27/3.27 | 0.00 |
| (521) | 3.05 | 3.04 | 0.01 |
| (530)/(433) | 2.86 | 2.86/2.86 | 0.00 |
| (442)/(600) | 2.78 | 2.78/2.78 | 0.00 |
| (611)/(532) | 2.71 | 2.70/2.70 | 0.01 |
| (620) | 2.64 | 2.64 | 0.00 |
| (541) | 2.58 | 2.57 | 0.01 |
| (622) | 2.51 | 2.51 | 0.00 |
| (631) | 2.46 | 2.46 | 0.00 |
| (444) | 2.41 | 2.41 | 0.00 |
| (550)/(710)/(543) | 2.36 | 2.36/2.36/2.36 | 0.00 |
| (640) | 2.31 | 2.31 | 0.00 |
| (721)/(552) | 2.27 | 2.27/2.27 | 0.00 |
| (642) | 2.23 | 2.23 | 0.00 |
| (730) | 2.19 | 2.19 | 0.00 |
| (732) | 2.12 | 2.12 | 0.00 |
| *a* = 16.67 nm | | | |

**Table S4.** Miller indices *(hkl)* and *d*-spacing of the diffraction peaks for the Cub_bi_/*I*23 phase observed at 60ºC in compound Di3-10.

| Di3-10 (*hkl*) | *d*_obs._ - spacing/nm | *d*_calc._ - spacing/nm | *d*_obs._ - *d*_calc._ |
| --- | --- | --- | --- |
| (310) | 5.02 | 5.01 | 0.01 |
| (222) | 4.58 | 4.58 | 0.00 |
| (321) | 4.24 | 4.24 | 0.00 |
| (400) | 3.96 | 3.96 | 0.00 |
| (330) | 3.74 | 3.74 | 0.00 |
| (420) | 3.54 | 3.54 | 0.00 |
| (422) | 3.23 | 3.24 | -0.01 |
| (431)/(510) | 3.11 | 3.11/3.11 | 0.00 |
| (521) | 2.89 | 2.89 | 0.00 |
| (530)/(433) | 2.72 | 2.72/2.72 | 0.00 |
| (442)/(600) | 2.64 | 2.64/2.64 | 0.00 |
| (611)/(532) | 2.57 | 2.57/2.57 | 0.00 |
| (620) | 2.51 | 2.51 | 0.00 |
| (541) | 2.44 | 2.45 | -0.01 |
| (622) | 2.39 | 2.39 | 0.00 |
| (631) | 2.34 | 2.34 | 0.00 |
| (444) | 2.29 | 2.29 | 0.00 |
| (550)/(710)/(543) | 2.24 | 2.24/2.24/2.24 | 0.00 |
| (640) | 2.20 | 2.20 | 0.00 |
| (721)/(552) | 2.16 | 2.16/2.16 | 0.00 |
| (642) | 2.12 | 2.12 | 0.00 |
| (730) | 2.08 | 2.08 | 0.00 |
| (732) | 2.01 | 2.01 | 0.00 |
| *a* = 15.85 nm | | | |

**Table S5.** Miller indices *(hkl)* and *d*-spacing of the diffraction peaks for the SmQ phase observed at 39ºC in compound Di3-12.

| Di3-12 (*hkl*) | *d*_obs._- spacing/nm | *d*_calc._ - spacing/nm | *d*_obs_*_._ - d*_calc._ |
| --- | --- | --- | --- |
| (110) | 8.55 | 8.55 | 0.00 |
| (112) | 6.52 | 6.52 | 0.00 |
| (004) | 5.04 | 5.04 | 0.00 |
| (114) | 4.33 | 4.34 | -0.01 |
| (220) | 4.24 | 4.27 | -0.03 |
| (222)/(301) | 3.94 | 3.94/3.95 | 0.00/-0.01 |
| (116) | 3.12 | 3.13 | -0.01 |
| (226) | 2.64 | 2.64 | 0.00 |
| (325)/(404) | 2.58 | 2.58/2.59 | 0.00/-0.01 |
| (334) | 2.48 | 2.48 | 0.00 |
| (431)/(501)/(118) | 2.41 | 2.40/2.40/2.42 | 0.01/0.01/-0.01 |
| (510)/(415) | 2.36 | 2.37/2.37 | -0.01/-0.01 |
| (521) | 2.23 | 2.23 | 0.00 |
| (336)/(228)/(327) | 2.17 | 2.17/2.17/2.18 | 0.00/0.00/-0.01 |
| *a* = *b* = 12.09 nm, *c* = 20.16 nm | | | |

**Table S6.** Miller indices *(hkl)* and *d*-spacing of the diffraction peaks for the SmQ phase observed at 63ºC in compound Di7-12.

| Di7-12 (*hkl*) | *d*_obs._- spacing/nm | *d*_calc._ - spacing/nm | *d*_obs_*_._ - d*_calc._ |
| --- | --- | --- | --- |
| (112) | 6.84 | 6.84 | 0.00 |
| (004) | 5.27 | 5.27 | 0.00 |
| (220) | 4.50 | 4.49 | 0.01 |
| (301) | 4.14 | 4.15 | 0.00 |
| (116) | 3.27 | 3.27 | 0.00 |
| (334) | 2.61 | 2.61 | 0.00 |
| (118) | 2.53 | 2.53 | 0.00 |
| *a* = *b* =12.71 nm, *c* = 21.06nm | | | |

**Table S7.** Miller indices *(hkl)* and *d*-spacing of the diffraction peaks for the $Ia\bar{3}d$ phase observed at 53ºC in compound Vin9-7NO_2_.

| Vin9-7NO_2_ (*hkl*) | *d*_obs._- spacing/nm | *d*_calc._ - spacing/nm | *d*_obs_*_._ - d*_calc._ |
| --- | --- | --- | --- |
| (211) | 3.33 | 3.33 | 0.00 |
| (220) | 2.88 | 2.88 | 0.00 |
| (321) | 2.18 | 2.18 | 0.00 |
| (400) | 2.04 | 2.04 | 0.00 |
| (420) | 1.82 | 1.82 | 0.00 |
| (332) | 1.74 | 1.74 | 0.00 |
| (422) | 1.66 | 1.66 | 0.00 |
| (431) | 1.60 | 1.60 | 0.00 |
| (521) | 1.49 | 1.49 | 0.00 |
| (440) | 1.43 | 1.44 | -0.01 |
| *a* = 8.15 nm | | | |

**Table S8.** Miller indices *(hkl)* and *d*-spacing of the diffraction peaks for the smectic phase observed at 141ºC in compound Si9-10.

| Si9-10 (*hkl*) | *d*_obs._- spacing/nm | *d*_calc._ - spacing/nm | *d*_obs_*_._ - d*_calc._ |
| --- | --- | --- | --- |
| (001) | 6.76 | 6.76 | 0.00 |
| (002) | 3.38 | 3.38 | 0.00 |
| *d* = 6.76 nm | | | |

**Table S9.** Miller indices *(hkl)* and *d*-spacing of the diffraction peaks for the Col_hex_ phase observed at 128ºC in compound Si9-12.

| Si9-12 (*hkl*) | *d*_obs._- spacing/nm | *d*_cal._ - spacing/nm | *d*_obs_*_._ - d*_calc._ |
| --- | --- | --- | --- |
| (10) | 7.02 | 7.02 | 0.00 |
| (11) | 4.05 | 4.05 | 0.00 |
| (20) | 3.51 | 3.51 | 0.00 |
| *a* = 8.10 nm | | | |

1. Additional calorimetric data





**Figure S6.** DSC thermograms of compound Vin7-10 on heating and cooling at rate of 5K·min^-1^. M = undetermined mesophase.

**Table S10.** Transition temperatures (*T*/℃) and associated enthalpy values [*∆H*/J g^-1^] of the investigated compounds as measured on heating and cooling by DSC.

| **Comd.** | **Heating**  T/^o^C [*ΔH*/J g^-1^] | **Cooling**  T/^o^C [*ΔH*/J g^-1^] |
| --- | --- | --- |
| **Vin7-12** | 1^st^: Cr 65[91.2] *I*23 70[1.6] Iso  2^nd^: 24[20.5] Cr 65[81.7] *I*23 72[0.1] Iso | Iso 70 Iso* 59[0.5] *I*23 34[68.6] Cr |
| **Vin9-7** | 1^st^: Cr 68[67.6] Cr 75[73.7] Iso  2^nd^: Cr 29[1.7] Cr 52[50.2] Cr 75[33.8] Iso | Iso 76 Iso* 46[24.1] Cr 5[7.2] Cr |
| **Vin9-7F** | 1^st^: Cr 69[57.1] Iso  2^nd^: Cr 47[38.9] Cr 72[26.0] Iso | Iso 60 Iso*29[0.9] Cr |
| **Vin9-7NO_2_** | 1^st^: Cr -5[2.8] Cr 58[34.7] $Ia\bar{3}d$ 91 [3.0] Iso  2^nd^: -3 *T*_g_ $Ia\bar{3}d$ 92[4.0] Iso | Iso 77[2.2] $Ia\bar{3}d$ *T*_g_ -1 |
| **Vin7-10** | 1^st^: Cr 20[5.0] Cr 56[69.1] M 64[0.6] Iso* 72 Iso  2^nd^: Cr -11[3.3] Cr 23[42.2] Cr 50[45.8] M 65[0.5] Iso* 73 Iso | Iso 72 Iso* 50[0.2] M -15[4.2] Cr |
| **Vin9-9** | 1^st^: Cr 14[3.3] Cr 62[0.3] Cr 73[67.7] Iso* 79 Iso  2^nd^: Cr -5[5.5] Cr 18[44.3] Cr 72[65.2] Iso* 79 Iso | Iso 77 Iso* 57[0.1] M -7 [2.5] Cr |
| **Di3-10** | 1^st^: Cr 58[27.8] Cr 68[2.2] *I*23 82 [2.3] Iso  2^nd^: 15 *T*_g_ *I*23 82[2.3] Iso | Iso 79 Iso* 74[0.8] *I*23 *T*_g_ 12 |
| **Di3-12** | 1^st^: Cr 60[12.6] Cr 78[51.2] M 83[2.2] Iso  2^nd^: SmQ+*I*23 72[1.4] M 84[2.4] Iso | Iso 77 Iso* 74[0.5] SmQ+*I*23 *T_g_* 12 |
| **Di7-12** | 1^st^: Cr 62[28.1] Cr 64[10.9] Cr 80[29.2] Iso  2^nd^: Cr 50[6.1] Cr 63[1.7] Cr 80[34.5] Iso | Iso 78 Iso* 71[0.60] SmQ+*I*23 33[2.6] Cr |
| **Si9-10** | 1^st^: *T*_g_ 16 G 53[0.8] X 127[0.6] Sm 163[0.3] Iso  2^nd^: *T*_g_ 15 M 117[0.1] X 127[0.3] Sm 163[0.1] Iso | Iso 159[0.1] Sm 124[0.2] X 111[0.1] M *T*_g_ 10 |
| **Si9-12** | 1^st^: *T*_g_ 15 G 53[0.5] Y 124[0.3] Col 143[0.2] Iso  2^nd^: *T*_g_ 14 Y 124[0.8] Col 143[0.2] Iso | Iso 136[0.3] Col 120[0.3] Y *T*_g_ 10 |

1. Additional chiroptical data





**Figure S7.** Optical Rotation (OR) of compound Vin7-12. **a.** Temperature dependence of brightness *vs* analyzer rotation angles with respect to 90° from polarizer as reference (from - 10° to +10° at 1° steps). Temperature range from 78 ^o^C to 52 ^o^C. Inset shows the expanded central region. OR was taken as analyzer angle of minimum brightness. **b.** Example fitting of brightness data with a negative Lorentzian at 68 ^o^C. R^2^ was above 0.99 for all temperatures.

1. Additional theory

As already described in the main text, we start by modifying the 1D Ising model, replacing the two spin states (up and down) of each lattice unit with three, i.e. left-handed (degeneracy 1), right-handed (degeneracy 1) and generic states (degeneracy *g*). Each lattice unit in the modified model would correspond to a molecular raft. We assume that, in isolation, all three states have the same energy 0. As in normal 1D Ising model, only nearest neighbor interaction is considered, and if two neighboring rafts are both left-handed or right-handed, the system energy is reduced by $\epsilon=k_{B}T_{\epsilon}$. The partition matrix for a pair of rafts can be written as

$V=\left( \begin{matrix} e^{\beta\epsilon} & 1 & g \\ 1 & e^{\beta\epsilon} & g \\ g & g & g^{2} \end{matrix} \right)$ (Eq. S1)

Here $\beta=\frac{1}{k_{B}T}$. The matrix *V* can be diagonalized to be

$PVP^{-1}=\left( \begin{matrix} A_{1} & 0 & 0 \\ 0 & A_{2} & 0 \\ 0 & 0 & A_{3} \end{matrix} \right)$ (Eq. S2)

The three eigenvalues of matrix *V*, i.e. *A*_1_, *A*_2_ and *A*_3_ are $\frac{\lambda+1+m\pm\sqrt{\left( \lambda+1-m \right)^{2}+8m}}{2}$ and $-1$ , respectively, where $\lambda=e^{\beta\epsilon}$, and $m=g^{2}$.

Assuming N rafts (units) are arranged in a 1-d loop, we have the system partition function

$Z_{N}=Tr\left( V^{N} \right)$ (Eq. S3)

The partition function is dominated by the largest eigenvalue $\frac{\lambda+1+m+\sqrt{\left( \lambda+1-m \right)^{2}+8m}}{2}$ and

$\ln Z_{N}=N\ln\frac{\lambda+1+m+\sqrt{\left( \lambda+1-m \right)^{2}+8m}}{2}$ (Eq. S4)

System energy is then

$E=-\frac{\partial lnZ_{N}}{\partial\beta}=-N\frac{\partial lnA}{\partial\beta}=-N\epsilon\lambda\frac{1+(\lambda+1-m)/\sqrt{\left( \lambda+1-m \right)^{2}+8m}}{\lambda+1+m+\sqrt{\left( \lambda+1-m \right)^{2}+8m}}$ (Eq. S5)

The energy can be rewritten as

$E=-N\epsilon\frac{\lambda}{\lambda-1}(\frac{1}{2}+\frac{\lambda-3-m}{2\sqrt{\left( \lambda+1-m \right)^{2}+8m}})$ (Eq. S6)

In the temperature range of interest $\lambda\gg1$, and the energy can be simplified as

$E\approx-N\epsilon(\frac{1}{2}+\frac{\lambda-3-m}{2\sqrt{\left( \lambda+1-m \right)^{2}+8m}})$ (Eq. S7)

The heat capacity $C_{p}$ can be expressed as

$C_{p}=k_{B}\frac{T_{\epsilon}^{2}}{T^{2}}\frac{2\lambda(\lambda+1+m)}{{[\left( \lambda+1-m \right)^{2}+8m]}^{3/2}}$ (Eq. S8)

$C_{p}$ maximum happens at $\lambda\sim m+3$, when *m* is sufficiently large. With this equation we can generate the $C_{p}$ hump which fits well the experimental data above the Iso-Iso* or Iso-LC transition, as shown below as an example for Di3-10 where we have used the following parameters: $\epsilon$ = 16.53 kJ mol^-1^, *g* = 15.5 and molar mass = 3750 g mol^-1^. The molar mass of the raft suggests it consists of ~2 dimers, or 4 monomers.

The probability $p$ of a raft unit being in the non-generic (either left- or right-handed) state can be calculated to be

$p=1-\frac{m}{\sqrt{\left( \lambda+1-m \right)^{2}+8m}}\cdot\frac{3+m-\lambda+\sqrt{\left( \lambda+1-m \right)^{2}+8m}}{\lambda+1+m+\sqrt{\left( \lambda+1-m \right)^{2}+8m}}$ (Eq. S9)

If the left- or right-handed blocks have on an average number <*n>* of rafts, system energy

$E=-pN\epsilon\frac{<n>-1}{<n>}$ (Eq. S10)

And

$<n>=\frac{pN\epsilon}{pN\epsilon+E}$ (Eq. S11)

As also laid out in the main text, we can consider that there is a critical $p$ value above which long range order will set in, whereby the weak interaction between rafts in neighboring 1-d strings will result in a further reduction of system’s energy. The reduction in energy, when the system is fully ordered ($p=1$), should be the interaction energy $\epsilon'$ between two chiral rafts in neighboring 1-d strings. We speculate that the effects of the introduction of weak interactions on our lattice model of the isotropic phase (Fig. 2C, main text) would be to reduce the system’s energy ($\Delta\epsilon$) as a function of probability $p$ according to a power law and

$\Delta\epsilon={\left( \frac{p-p_{c}}{1-p_{c}} \right)^{\delta}\epsilon}^{'} p_{c}<p<1$ (Eq. S12)

This would have a consequence of changing the value of$\lambda$ to $e^{\beta(\epsilon+\Delta\epsilon)}$, and by iteration we can arrive at a set of self-consistent $p$, $\lambda$, $E$ and $C_{p}$ values at different temperatures. The average number of rafts in chiral blocks, after introduction of weak interactions, becomes

$<n>=\frac{pN(\epsilon+\Delta\epsilon)}{pN\left( \epsilon+\Delta\epsilon\right)+E}$ (Eq. S13)

The calculated average size of chiral blocks for Di3-10 at different temperatures, with and without considering the weak interactions, are shown in Figure S6. At the Iso-Iso* transition the average number of rafts in chiral blocks is 26. As the π-π stacking distance is ~0.45nm, the average block size at Iso-Iso* transition is ~12nm. At $C_{p}$ maximum, <n> is ~13, or block size ~ 6 nm.

In Figure S8 we also present the effect of the change in ratio of energies of the weak interactions *ε’* to that of the strong interactions *ε* on the shape of the *C_p_* curve.

Figure S8. Heat capacity curves calculated for three different ε’/ε ratios displayed on two different *C_p_* scales.

1. Additional comparison of theory and experiments


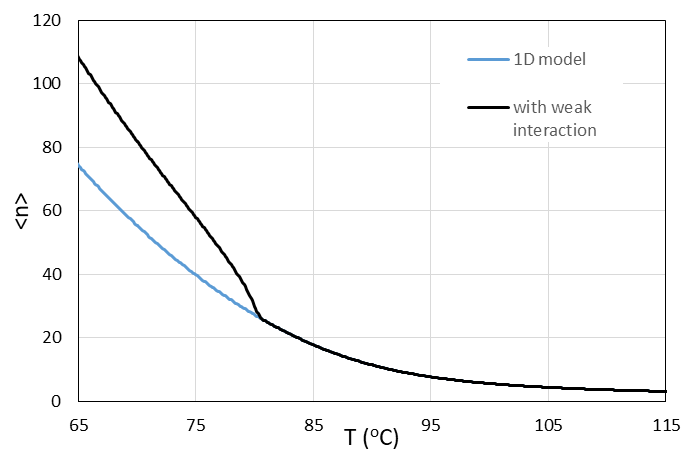


**Figure S9.** Average number of molecules in a cluster as a function of temperature, calculated from best-fit models for Di3-10.


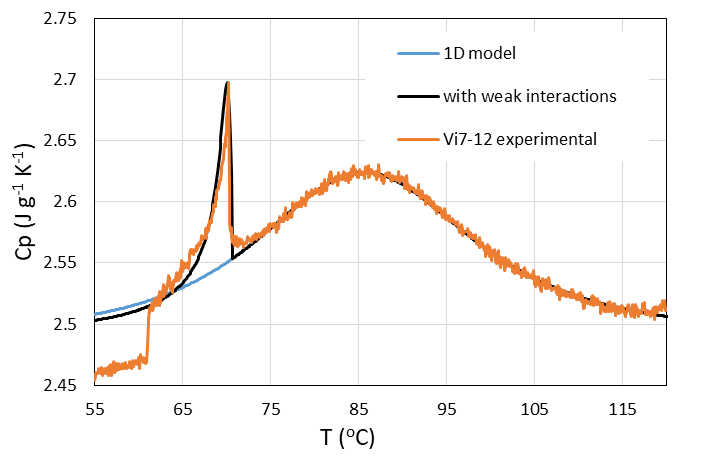


**Figure S10.** Comparison of reversing $C_{p}$ data of Vin7-12 obtained by MDSC and those calculated from best-fit models. Orange line: experimental; blue line: 1D model; black line: weak interactions included.


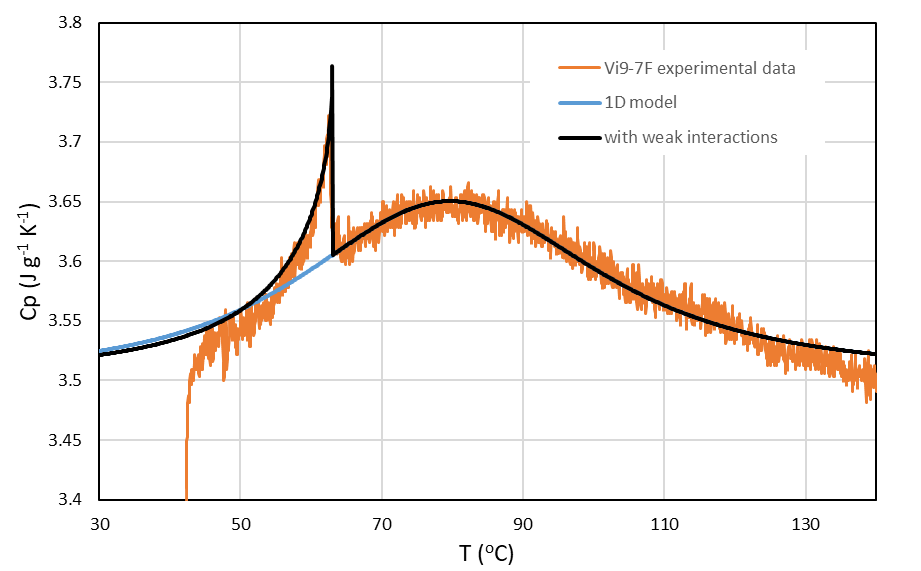


**Figure S11.** Comparison of reversing $C_{p}$ data of Vin9-7F obtained by MDSC and those calculated from best-fit models. Orange line: experimental; blue line: 1D model; black line: weak interactions included.


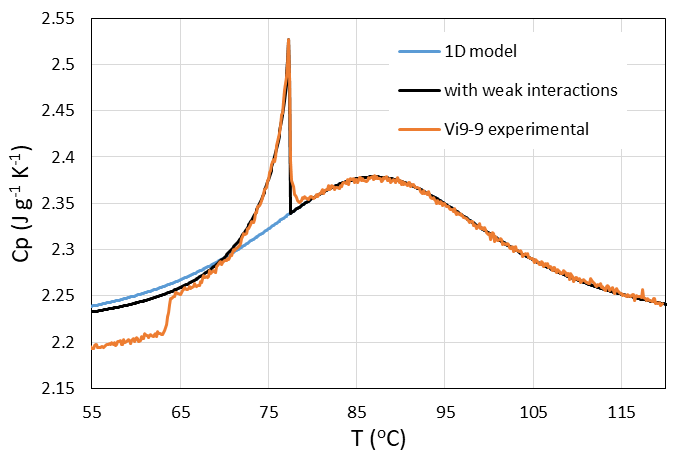


**Figure S12.** Comparison of reversing $C_{p}$ data of Vin9-9 and those calculated from best-fit models. Orange line: experimental; blue line: 1D model; black line: weak interactions included.


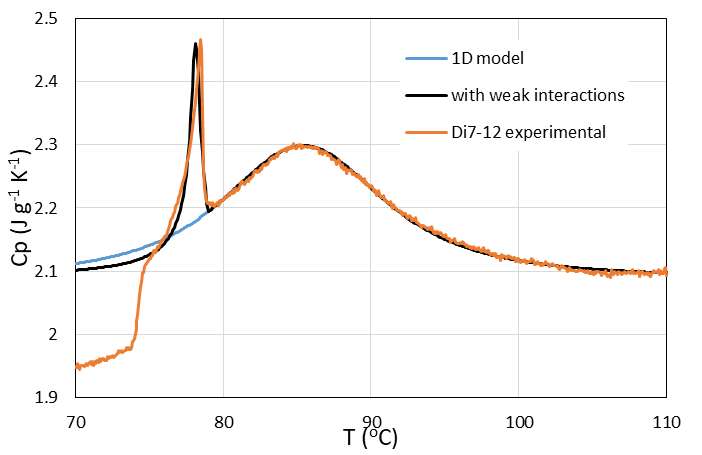


**Figure S13.** Comparison of reversing $C_{p}$ data of Di7-12 and those calculated from best-fit models. Orange line: experimental; blue line: 1D model; black line: weak interactions included.


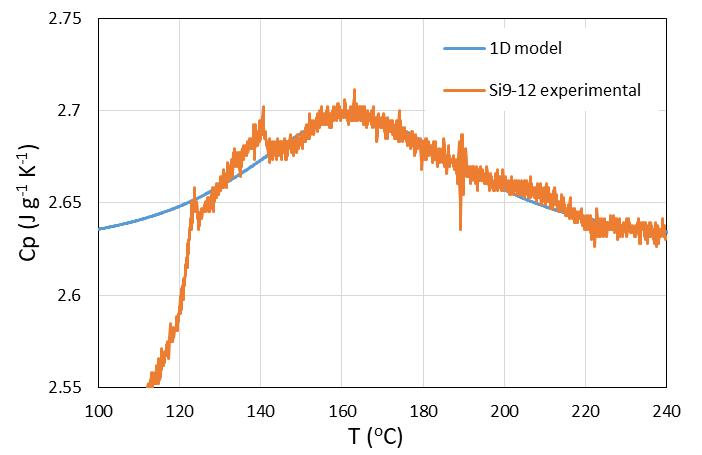


**Figure S14.** Comparison of reversing $C_{p}$ data of Si9-12 and those calculated from best-fit models. Orange line: experimental; blue line: 1D model.

1. Methods of physical and structural characterization

**DSC thermograms** were recorded with a TA Instruments DSC250 at a rate of 5K·min^-1^. Prior to filling with sample, the thermograms of the empty pan with lid were recorded in heating and cooling and used as baseline for the subsequently recorded sample thermograms. All thermograms shown are baseline-subtracted. Temperature was calibrated using melting of indium and ice of distilled water, using a series of heating rates. Extrapolation to negative rates was used for calibration of cooling thermograms. **Modulated DSC** thermograms were recorded on the same instrument; the linear cooling/heating rate was 0.04 K.min^-1^, the temperature oscillation amplitude was 0.07 K with an oscillation period of 20 s. Empty pan baseline was subtracted, as above. Heat capacity was calibrated with a sapphire standard.

**Synchrotron small-angle X-ray powder diffraction** experiments were performed on beamline BL16B1 at Shanghai Synchrotron Radiation Facility (SSRF), and at beamline I22 of Diamond Light Source, U.K. In both cases samples were held in Lindemann glass capillaries in a modified Linkam heating cell with mica windows. During X-ray exposure the capillary was rotating at ca. 100 rpm in order to minimize the effect of large LC domains, temperature gradients and radiation damage. Determination of coherence length from SAXS curves is described in Figures S4 and S5 and the accompanying text in SI. **Optical rotation** **measurement** is described in Figure S7. **CD experiments** were carried out at station B23, Diamond Light Source, using synchrotron light deflected vertically and the thin film sample sandwiched between fused silica plates lying horizontally in a Linkam heating cell.

**Optical micrographs** of mesophases with crossed polarizers were recorded on an Olympus BX-51 microscope equipped with a Linkam LTS420 hot stage and a T95-HS controller. **Freeze-fracture microtomy** was performed with a Balzers BAF 400 T instrument. Coating was done by evaporating platinum, then carbon. Replicas wer observed with a Jeol JEM-2100 Plus transmission electron microscope at 200 kV accelerating voltage. Prior to crosslinking in the Iso* state, the sample for **atomic force microscopy** was spin-coated on a highly ordered pyrolytic graphite substrate. AFM imaging was done in tapping mode on a Bruker Multimode 8 instrument with Nanoscope V controller.

1. Synthesis and analytical data

***General remarks***

Methyl 3,4,5-trihydroxybenzoate (methyl gallate, 98%), 1-bromododecane (98.5%), allyl bromide (98%), methyl 4-hydroxybenzoate (99%), 4,4’-dihydroxybiphenyl (98%), 1,1,3,3-tetramethyldisiloxane (98%), polymethylhydrosiloxane (trimethylsilyl terminated, M_n_,_GPC_=3200 g mol^-1^, PDI=2.43). All other chemicals or solvents were of analytical grade and were used as received. Silica gel (Qingdao Ocean Chemical Co., Ltd) were used as received. Dry DCM was dried by refluxed at least 12 hours with calcium hydride and stored with activated standard 3A Grade. Dry THF and dry DMF were ordered from Beijing InnoChem Science & Technology Co., Ltd.

***Abbreviations***

PMHS - polymethylhydrosiloxane (trimethylsilyl terminated); DCM - dichloromethane; DMF - dimethylformamide; EtOAc - ethyl acetate; PE - petroleum ether (fraction 60-90); THF - tetrahydrofuran; MeCN - acetonitrile, CHCl_3_ - chloroform.

**Synthesis and Methods of Chemical Analysis**

Reactions requiring an inert gas atmosphere were conducted under nitrogen. Dry dichloromethane (DCM) was dried by reflux at least 12 hours with calcium hydride and stored with activated standard 3A grade molecular sieve. Dry tetrahydrofuran (THF) and dry dimethylformamide (DMF) were obtained from Beijing InnoChem Science & Technology Co., Ltd. All other chemicals and solvents were of analytical grade and were used as received. Column chromatography was performed with silica gel (300-400 mesh) produced by Qingdao Ocean Chemical Co., Ltd.

^1^H-NMR spectra, ^13^C-NMR spectra, Correlated spectroscopy (^1^H-^1^H COSY), Heteronuclear Single-Quantum Correlation spectroscopy (HSQC) and Heteronuclear Multiple Bond Coherence spectroscopy (HMBC) were recorded on a Bruker Advance III HD 600 spectrometer using chloroform-d (CDCl_3_) and dimethyl sulfoxide-d_6_ (DMSO-d_6_) as solvents and tetramethyl silane (TMS) as internal standard at 298K. Chemical shifts are reported as ppm. Purity of products was determined by a combination of techniques including thin-layer chromatography (TLC) on silica gel-coated glass plates and elemental analysis using Elementar UNICUBE. Mass spectra were recorded on a quadrupole-orbitrap mass spectrometer (QExactive, ThermoFisher Scientificl, APCI: spray-voltage 5kV, solvent: MeCN for Vin monomers and mixture of MeCN and THF for Di dimers, sheath gas nitrogen). The gel permeation chromatographic (GPC) analysis for compound Si9-10 and Si9-12 was performed on a Waters 1515-2707-2414 instrument, solvent CHCl_3_, flow rate 1mL/min, temperature 35°C, using polystyrene standards. GPC analysis for polymethylhydrosiloxane (trimethylsilyl terminated, PMHS) was carried out on an Agilent 1260 Infinity II instrument, solvent CHCl_3_, flow rate 0.6 mL/min, temperature 50°C, with polymethyl methacrylate as standard.

**Scheme S1.** Reagent and conditions for synthesis of compounds: *i*) K_2_CO_3_, dry DMF, 80 ^o^C, 24 hrs; *ii*) KOH, ethyl alcohol, 80 °C, 12 hrs; *iii*) K_2_CO_3_, acetone, 80 °C, 24 hrs; *iv*) DMAP, EDC, dry DMF, 0 ^o^C for 2 hrs and then 48 hrs at r.t.; *v*) Karstedt’s catalyst, r.t. /60^o^C, 48hrs, dry toluene.

**9.1 General procedures for the synthesis of compounds**

The compound Vins and Polymers were prepared according to the literature,^S^^[[4]](#endnote-4),S^^[[5]](#endnote-5)^ the procedures are described below.

*Procedure i (Alkyl bromide substitution, Williamson Ether Synthesis):*

A mixture containing benzene hydroxyl (1 eq.), anhydrous K_2_CO_3_ (5 eq.) were purged with nitrogen. Bromoalkanes (5 eq.) and dry DMF were added and the solution was refluxed for 24 hours. After the reaction reached room temperature, H_2_O was added and hydrochloric acid was added until pH to 5, then it was extracted with EtOAc. The organic layer was dried with anhydrous Mg_2_SO_4_, filtered and concentrated. The crude product was purified by column chromatography.

*Procedure ii (hydrolysis):*

A mixture containing ester-based compound 3 or 7 (1 eq.) and KOH (5 eq.) in ethanol was refluxed for 12 hours and allowed to cool to room temperature. The solution was acidified with HCl, then precipitated into ice H_2_O and chilled for 24 hours. The solid was filtered and dried under vacuum.

*Procedure iii (Alkene bromide substitution, Williamson Ether Synthesis):*

A mixture containing benzene hydroxyl (1 eq.), anhydrous K_2_CO_3_ (4 eq.) were purged with nitrogen. Alkene bromide (1.1 eq.) and acetone were added and the solution was refluxed for 16 hours. After the reaction reached room temperature, it was extracted with EtOAc. The organic layer was dried with anhydrous Mg_2_SO_4_, filtered and concentrated. The crude product was purified by column chromatography.

*Procedure iv (Esterification):*

Carboxylic acid (1eq.), hydroxy compound (1.05 eq.), DMAP (1.5 eq.) and EDC (1.5 eq.) were resolved in dry DCM on nitrogen atmosphere. Then the system was stirred 48 hours at room temperature. The mixture was extracted with DCM. The volume of the DCM was reduced on the rotary evaporator. It is purified by column chromatograph.

*Procedure v (Hydrosilylation):*

Double bond compound (1.1 eq.), poly(hydrogenmethylsiloxane) (PHMS, Mn=3200 g·mol^-1^, PDI=2.43) (Si-H:1 eq.) and 43ppm Karstedt’s catalyst were dissolved in 5 mL dry toluene. The reaction mixture was heated for 48 hours at 60 ℃ and purged with nitrogen. The solution was then cooled and precipitated into methanol. The precipitate was filtered, dissolved in CH_2_Cl_2_, and precipitated in methyl alcohol and acetone cooled below to -10 ℃ twice, filtered, and dried to yield white solid.

*Synthesis of compound 4*

According to procedure *i*: A mixture of the methyl 3,4,5-trihydroxybenzoate (1 eq.), anhydrous K_2_CO_3_ (5 eq.) were purged with nitrogen. Bromoalkanes (5 eq.) and dry DMF were added and the solution was refluxed for 16 hours. After the reaction reached room temperature, it was extracted with EtOAc. The organic layer was dried with anhydrous Mg_2_SO_4_, filtered and concentrated. The crude product was purified by column chromatography, the ratio of solvent PE/EtOAc is 5/1 for TLC, R_f_ is about 0.5. Compound 3 was hydrolyzed to product compound 4 with procedure *ii*.

*4C7: 3,4,5-tris(heptyloxy)benzoic acid, yield 87 %, white solid.*

^1^H NMR (600 MHz, CDCl_3_) δ 7.32 (s, 2H, Ar-*H*), 4.03 (dt, *J* = 11.4, 6.6 Hz, 6H, OC*H_2_* CH_2_), 1.98 – 1.65 (m, 6H, OCH_2_C*H_2_*), 1.55 – 1.43 (m, 6H, OCH_2_CH_2_C*H_2_*), 1.42 – 1.20 (m, 18H, C*H_2_*), 0.89 (td, *J* = 6.9, 3.4 Hz, 9H, C*H_3_*).

*4C9:* *3,4,5-tris(nonyloxy)benzoic acid, yield 95 %, white solid.*

^1^H NMR (600 MHz, CDCl3) δ 7.33 (s, 2H, Ar-*H*), 4.04 (dq, *J* = 13.8, 7.1, 6.7 Hz, 6H, OC*H_2_* CH_2_), 1.79 (dq, *J* = 41.1, 7.6 Hz, 6H OCH_2_C*H_2_*), 1.57 – 1.42 (m, 6H, OCH_2_CH_2_C*H_2_*), 1.32 (ddt, J = 28.1, 12.9, 7.0 Hz, 30H, C*H_2_*), 0.89 (q, J = 7.5 Hz, 9H, C*H_3_*).

*4C10: 3,4,5-tris(decyloxy)benzoic acid, yield 85 %, white solid.*

^1^H NMR (600 MHz, CDCl_3_) δ 7.33 (s, 2H, Ar-*H*), 4.22 – 3.82 (m, 6H, OC*H_2_*CH_2_), 1.94 – 1.65 (m, 6H, OCH_2_C*H_2_*), 1.56 – 1.43 (m, 6H, OCH_2_CH_2_C*H_2_*), 1.42 – 1.18 (m, 36H, C*H_2_*), 0.88 (t, *J* = 6.9 Hz, 9H, C*H_3_*).

*4C12: 3,4,5-tris(dodecyloxy)benzoic acid, yield 88%, white solid.*

^1^H NMR (600 MHz, CDCl_3_) δ 7.32 (s, 2H, Ar-*H*), 4.03 (dt, *J* = 12.7, 6.5 Hz, 6H, OC*H_2_*CH_2_), 1.79 (ddd, *J* = 36.7, 14.5, 7.2 Hz, 6H, OCH_2_C*H_2_*), 1.60 – 1.40 (m, 6H, OCH_2_CH_2_C*H_2_*), 1.38 – 1.09 (m, 48H, C*H_2_*), 0.88 (t, *J* = 7.0 Hz, 9H, C*H_3_*).

*Synthesis of compound 8*

According to procedure *iii*: A mixture containing compound 6 (1 eq.), anhydrous K_2_CO_3_ (4 eq.) were purged with N_2_. Bromoalkanes (1.1 eq.) and acetone were added and the solution was refluxed for 16 hours. After the reaction reached room temperature, it was extracted with EtOAc. The organic layer was dried with anhydrous Mg_2_SO_4_, filtered and concentrated. The crude product was purified by column chromatography to obtain compound 7, the ratio of solvent PE/DCM is 1/4 for TLC, R_f_ is about 0.6. According to procedure *ii*: Compound 7 was hydrolyzed to product compound 8.

*8C3: 4-(allyloxy) benzoic acid, yield 95%, white solid.*

^1^H NMR (600 MHz, CDCl_3_) δ 8.28 – 7.84 (m, 2H, Ar-*H*), 6.96 (dd, *J* = 6.7, 4.9 Hz, 2H, Ar-*H*), 6.06 (ddt, *J* = 17.2, 10.6, 5.3 Hz, 1H, =C*H*), 5.44 (dd, *J* = 17.3, 1.4 Hz, 1H, =C*H_a_*H_b_), 5.33 (dd, *J* = 10.5, 1.3 Hz, 1H, =CH_a_*H_b_*), 4.78 – 4.43 (m, 2H, C*H_2_*).

*8C7: 4-(hept-6-en-1-yloxy) benzoic acid, yield 85%, white solid.*

^1^H NMR (600 MHz, CDCl_3_) δ 8.05 (d, *J* = 8.4 Hz, 2H, Ar-*H*), 6.93 (d, *J* = 8.4 Hz, 2H, Ar-*H*), 5.82 (dq, *J* = 10.1, 6.7 Hz, 1H, =C*H*), 5.02 (d, *J* = 17.1 Hz, 1H, =C*H_a_*H_b_), 4.96 (d, *J* = 10.2 Hz, 1H, =CH_a_*H_b_*), 4.03 (t, *J* = 6.5 Hz, 2H, OC*H_2_*), 2.30 - 1.95 (m, 2H, =CHC*H_2_*), 1.82 (dd, *J* = 13.2, 6.6 Hz, 2H, OCH_2_C*H_2_*), 1.65 - 1.34 (m, 4H, C*H_2_*).

*8C9: 4-(non-8-en-1-yloxy) benzoic acid, yield 94%, white solid.*

^1^H NMR (600 MHz, CDCl_3_) δ 8.05 (d, *J* = 8.7 Hz, 2H, Ar-*H*), 6.93 (d, *J* = 8.7 Hz, 2H, Ar-*H*), 5.81 (ddt, *J* = 16.9, 10.2, 6.7 Hz, 1H, =C*H*), 5.00 (d, *J* = 17.1 Hz, 1H, =C*H_a_*H_b_), 4.94 (d, *J* = 10.1 Hz, 1H, =CH_a_*H_b_*), 4.02 (t, *J* = 6.5 Hz, 2H, OC*H_2_*), 2.05 (q, *J* = 6.9 Hz, 2H, =CHC*H_2_*), 1.90 - 1.67 (m, 2H, OCH_2_C*H_2_*), 1.57 - 1.44 (m, 2H, OCH_2_CH_2_C*H_2_* ), 1.44 - 1.18 (m, 6H, C*H_2_*).

*8C9-F: 3-fluoro-4-(non-8-en-1-yloxy) benzoic acid, yield 87%, white solid.*

^1^H NMR (600 MHz, CDCl_3_) δ 7.87 (d, *J* = 8.5 Hz, 1H, *p*F-Ar-*H*), 7.80 (d, *J* = 11.5 Hz, 1H,  *o*F-Ar-*H*), 6.99 (t, *J* = 8.3 Hz, 1H, *m*F-Ar-*H*), 5.81 (ddt, *J* = 16.9, 10.2, 6.7 Hz, 1H, =C*H*), 5.00 (d, *J* = 17.1 Hz, 1H, =C*H_a_*H_b_), 4.94 (d, *J* = 10.1 Hz, 1H, =CH_a_*H_b_*), 4.10 (t, *J* = 6.5 Hz, 2H, OC*H_2_*), 2.05 (q, *J* = 6.8 Hz, 2H, =CHC*H_2_*), 1.96 - 1.70 (m, 2H, OCH_2_C*H_2_*), 1.61 - 1.44 (m, 2H, OCH_2_CH_2_C*H_2_*), 1.44 - 1.27 (m, 6H, C*H_2_*).

*8C9-NO2: 3-nitro-4-(non-8-en-1-yloxy) benzoic acid, yield 51%, white sold.*

^1^H NMR (600 MHz, CDCl_3_) δ 8.55 (d, *J* = 2.0 Hz, 1H, *o*NO_2_-Ar-*H*), 8.24 (dd, *J* = 8.8, 2.0 Hz, 1H,  *p*NO_2_-Ar-*H*), 7.14 (d, *J* = 8.9 Hz, 1H, *m*NO_2_-Ar-*H*), 5.92 – 5.72 (m, 1H, =C*H*), 5.00 (dd, *J* = 17.1, 1.5 Hz, 1H, =C*H_a_*H_b_), 4.94 (d, *J* = 10.0 Hz, 1H, =CH_a_*H_b_*), 4.19 (t, *J* = 6.4 Hz, 2H, OC*H_2_*), 2.05 (q, *J* = 6.9 Hz, 2H, =CHC*H_2_*), 1.93 - 1.80 (m, 2H, OCH_2_C*H_2_*), 1.54 - 1.30 (m, 8H, C*H_2_*).

*Synthesis of intermediate compound 10*

According to procedure *iv*: A solution of compound 8 (1 equ), compound 9 (5 equ), EDC (1.5 equ), DMAP (0.5 equ) in dry DMF was stirred at 0℃. Then the system was stirred 24 hours at room temperature on nitrogen. The mixture was extracted with EtOAc. The volume of the EtOAc was reduced on the rotary evaporator and then the obtained solid recrystallized from ethanol three times. Then was purified on column chromatography the ratio of solvent DCM/EtOAc is 10/1 for TLC, R_f_ is about 0.7. A white powder was obtained.

*10C3: 4'-hydroxy-[1,1'-biphenyl]-4-yl 4-(allyloxy) benzoate, yield 52%, white solid.*

^1^H NMR (600 MHz, DMSO-*d*_6_) δ 9.58 (s, 1H, O*H*), 8.09 (d, *J* = 8.9 Hz, 2H, Ar-*H*), 7.64 (d, *J* = 8.6 Hz, 2H, Ar-*H*), 7.51 (d, *J* = 8.6 Hz, 2H, Ar-*H*), 7.29 (d, *J* = 8.6 Hz, 2H, Ar-*H*), 7.15 (d, *J* = 8.9 Hz, 2H, Ar-*H*), 6.86 (d, *J* = 8.6 Hz, 2H, Ar-*H*), 6.08 (ddd, *J* = 22.5, 10.5, 5.2 Hz, 1H, =C*H*), 5.44 (dd, *J* = 17.3, 1.6 Hz, 1H, =C*H_a_*H_b_), 5.31 (dd, *J* = 10.6, 1.4 Hz, 1H, =CH_a_*H_b_*), 4.71 (d, *J* = 5.2 Hz, 2H, =CHC*H_2_*).

*10C7: 4'-hydroxy-[1,1'-biphenyl]-4-yl 4-(hept-6-en-1-yloxy) benzoate, yield 40%, white solid.*

^1^H NMR (600 MHz, DMSO-*d*_6_) δ 9.57 (s, 1H, O*H*), 8.08 (d, *J* = 7.3 Hz, 2H, Ar-*H*), 7.64 (d, *J* = 7.1 Hz, 2H, Ar-*H*), 7.51 (d, *J* = 7.2 Hz, 2H, Ar-*H*), 7.28 (d, *J* = 7.1 Hz, 2H, Ar-*H*), 7.11 (d, *J* = 7.5 Hz, 2H, Ar-*H*), 6.86 (d, *J* = 7.9 Hz, 2H, Ar-*H*), 5.82 (tt, *J* = 10.6, 6.2 Hz, 1H, =C*H*), 5.02 (d, *J* = 17.2 Hz, 1H, =C*H_a_*H_b_), 4.96 (d, *J* = 10.2 Hz, 1H, =CH_a_*H_b_*), 4.09 (t, *J* = 6.2 Hz, 2H, OC*H_2_*), 2.06 (d, *J* = 5.8 Hz, 2H, =CHC*H_2_*), 1.75 (d, *J* = 6.1 Hz, 2H, OCH_2_C*H_2_*), 1.43 (s, 4H, C*H_2_*).

*10C9: 4'-hydroxy-[1,1'-biphenyl]-4-yl 4-(non-8-en-1-yloxy) benzoate, yield 47%, white solid.*

^1^H NMR (600 MHz, DMSO-*d*_6_) δ 9.56 (s, 1H, O*H*), 8.08 (d, *J* = 8.7 Hz, 2H, Ar-*H*), 7.64 (d, *J* = 8.5 Hz, 2H, Ar-*H*), 7.51 (d, *J* = 8.5 Hz, 2H, Ar-*H*), 7.28 (d, *J* = 8.5 Hz, 2H, Ar-*H*), 7.12 (d, *J* = 8.7 Hz, 2H, Ar-*H*), 6.86 (d, *J* = 8.5 Hz, 2H, Ar-*H*), 5.80 (ddd, *J* = 17.1, 6.7, 3.5 Hz, 1H, =C*H*), 5.00 (d, *J* = 17.2 Hz, 1H, =C*H_a_*H_b_), 4.94 (d, *J* = 10.2 Hz, 1H, =CH_a_*H_b_*), 4.09 (t, *J* = 6.4 Hz, 2H, OC*H_2_*), 2.02 (q, *J* = 6.9 Hz, 2H, =CHC*H_2_*), 1.87 - 1.65 (m, 2H, OCH_2_C*H_2_*), 1.55 - 1.40 (m, 2H, OCH_2_CH_2_C*H_2_* ), 1.40 - 1.18 (m, 6H, C*H_2_* ).

*10C9F: 4'-hydroxy-[1,1'-biphenyl]-4-yl 3-fluoro-4-(non-8-en-1-yloxy) benzoate, yield 38%, white solid.*

^1^H NMR (600 MHz, DMSO-*d*_6_) δ 9.57 (s, 1H, O*H*), 7.96 (d, *J* = 8.7 Hz, 1H, *p*F-Ar-*H*), 7.89 (d, *J* = 11.6 Hz, 1H, *o*F-Ar-*H*), 7.65 (d, *J* = 8.4 Hz, 2H, Ar-*H*), 7.51 (d, *J* = 8.4 Hz, 2H, Ar-*H*), 7.38 (t, *J* = 8.5 Hz, 1H, *o*F-Ar-*H*), 7.30 (d, *J* = 8.4 Hz, 2H, Ar-*H*), 6.86 (d, *J* = 8.5 Hz, 2H, Ar-*H*), 5.80 (d, *J* = 6.8 Hz, 1H, =C*H*), 5.00 (d, *J* = 17.2 Hz, 1H, =C*H_a_*H_b_), 4.94 (d, *J* = 10.2 Hz, 1H, =C*H_a_*H_b_), 4.18 (t, *J* = 6.4 Hz, 2H, OC*H_2_*), 2.02 (q, *J* = 6.7 Hz, 2H, =CHC*H_2_*), 1.84 - 1.62 (m, 2H, OCH_2_C*H_2_*), 1.52 - 1.40 (m, 2H, OCH_2_CH_2_C*H_2_*), 1.40 - 1.22 (m, 6H, C*H_2_*).

*10C9NO_2_: 4'-hydroxy-[1,1'-biphenyl]-4-yl 3-nitro-4-(non-8-en-1-yloxy) benzoate, yield 33%, white solid.*

^1^H NMR (600 MHz, DMSO-*d*_6_) δ 8.57 (d, *J* = 2.2 Hz, 1H, *o*NO_2_-Ar-*H*), 8.36 (dd, *J* = 8.9, 2.1 Hz, 1H, *p*NO_2_-Ar-*H*), 7.66 (d, *J* = 8.6 Hz, 2H, Ar-*H*), 7.58 (t, *J* = 9.8 Hz, 1H, *m*NO_2_-Ar-*H*), 7.50 (dd, *J* = 25.6, 8.7 Hz, 2H, Ar-*H*), 7.33 (d, *J* = 8.6 Hz, 2H, Ar-*H*), 6.84 (dd, *J* = 18.5, 7.8 Hz, 2H, Ar-*H*), 5.80 (ddt, *J* = 16.9, 10.2, 6.7 Hz, 1H, =C*H*), 5.00 (dd, *J* = 17.2, 1.7 Hz, 1H, =C*H_a_*H_b_), 4.94 (d, *J* = 10.1 Hz, 1H, =CH_a_*H_b_*), 4.36 (t, *J* = 5.1 Hz, 2H, , OC*H_2_*CH_2_), 2.02 (dd, *J* = 13.9, 6.9 Hz, 2H, =CHC*H_2_*), 1.83 - 1.69 (m, 2H, , OCH_2_C*H_2_*), 1.50 - 1.40 (m, 2H, CH_2_, OCH_2_CH_2_C*H_2_*), 1.40 - 1.28 (m, 6H, C*H_2_*).

*Synthesis of compound Vin m-n*

According to procedure *iv*: Compound 4 (1equ), compound 10 (1.05 eq.), DMAP (1.5 eq.) and EDC (1.5 eq.) resolved in dry DCM. Then the system was stirred 48 hours at room temperature. The mixture was extracted with DCM. The volume of the DCM was reduced on the rotary evaporator. It is purified by column chromatograph, the ratio of solvent PE/DCM is 1/2 for TLC, R_f_ is about 0.5.

*Vin3-10: 4'-((4-(allyloxy) benzoyl) oxy)-[1,1'-biphenyl]-4-yl-3,4,5tris(decyloxy)benzoate, yield 89%, white solid.*

^1^H NMR (600 MHz, CDCl_3_) δ 8.18 (d, *J* = 8.9 Hz, 2H, Ar-*H*), 7.64 (d, *J* = 8.3 Hz, 4H, Ar-*H*), 7.43 (s, 2H, Ar-*H*), 7.28 (t, *J* = 8.1 Hz, 4H, Ar-*H*), 7.02 (d, *J* = 8.9 Hz, 2H, Ar-*H*), 6.08 (ddt, *J* = 17.2, 10.5, 5.3 Hz, 1H, -C*H*=), 5.46 (dd, *J* = 17.3, 1.4 Hz, 1H, =C*H_a_*H_b_), 5.35 (dd, *J* = 10.5, 1.3 Hz, 1H, =CH_a_*H_b_*), 4.64 (dd, *J* = 3.9, 1.3 Hz, 2H, OC*H_2_*CH), 4.06 (q, *J* = 6.3 Hz, 6H, OC*H_2_*CH_2_), 2.05 - 1.66 (m, 6H, OCH_2_C*H_2_*), 1.53 - 1.43 (m, 6H, OCH_2_CH_2_C*H_2_*), 1.42 - 1.07 (m, 36H, C*H_2_*), 0.88 (td, *J* = 7.0, 4.1 Hz, 9H, C*H_3_*). ^13^C NMR (151 MHz, CDCl_3_) δ 165.1, 164.9 (*C*=O), 163.0, 153.0, 150.6, 150.5, 143.0 (*C_Ar_*-O), 138.2, 138.1(*C_Ar_*-*C_Ar_*), 132.4 (*C*H=), 132.4, 128.2, 128.2 (*C_Ar_*-H), 123.9 (*C_Ar_*-COO-Ar), 122.2, 122.1 (*C_Ar_*-H), 121.9 (*C_Ar_*-COO-Ar), 118.3 (*C*H_2_=), 114.6, 108.6, 104.8 (*C_Ar_*-H), 73.6, 69.3, 69.0 (Ar-O*C*H_2_), 32.0, 31.9, 30.4, 29.7, 29.7, 29.6, 29.6, 29.6, 29.4, 29.4, 29.3, 26.1, 26.1 (*C*H_2_), 22.7, 22.7 (*C*H_2_CH_3_), 14.1(*C*H_3_),. HRMS (ESI, MeCN): calc. for [M+H] ^+^ (*m/z)*: 919.6054; Found: 919.6083.

*Vin3-12: 4'-((4-(but-3-en-1-yloxy) benzoyl) oxy)-[1,1'-biphenyl]-4-yl3,4,5-tris(dodecyloxy)-benzoate, yield 87%, white solid.*

^1^H NMR (600 MHz, CDCl_3_) δ 8.18 (d, *J* = 8.8 Hz, 2H, Ar-*H*), 7.64 (d, *J* = 8.4 Hz, 4H, Ar-*H*), 7.43 (s, 2H, Ar-*H*), 7.28 (dd, *J* = 14.9, 6.8 Hz, 6H, Ar-*H*), 7.02 (d, *J* = 8.8 Hz, 2H, Ar-*H*), 6.07 (dd, *J* = 11.3, 5.9 Hz, 1H, -C*H*=), 5.46 (d, *J* = 17.3 Hz, 1H, =C*H_a_*H_b_), 5.35 (d, *J* = 10.5 Hz, 1H, =CH_a_*H_b_*), 4.65 (d, *J* = 5.2 Hz, 2H, OC*H_2_*CH), 4.06 (q, *J* = 6.3 Hz, 6H, OC*H_2_*CH_2_), 1.81 (ddd, *J* = 36.2, 14.5, 7.2 Hz, 6H, OCH_2_C*H_2_*), 1.49 (dt, *J* = 15.2, 7.6 Hz, 6H, OCH_2_CH_2_C*H_2_*), 1.41 - 1.18 (m, 48H, C*H_2_*), 0.88 (td, *J* = 6.8, 3.4 Hz, 9H, C*H_3_*). ^13^C NMR (151 MHz, CDCl_3_) δ 165.1, 164.9 (*C*=O), 163.0, 153.0, 150.6, 150.5, 143.0 (*C_Ar_*-O), 138.2 , 138.1(*C_Ar_*-*C_Ar_*), 132.4 (*C*H=), 132.4, 128.2, 128.2 (*C_Ar_*-H), 124.8, 123.9 (*C_Ar_*-COO-Ar), 122.2, 122.1 (*C_Ar_*-H), 121.9 (*C_Ar_*-COO-Ar), 118.3 (*C*H_2_=), 114.6, 108.6 (*C_Ar_*-H), 73.6, 69.3, 69.0 (Ar-O*C*H_2_), 32.0, 31.9, 30.4, 29.8, 29.8, 29.7, 29.7, 29.6, 29.6, 29.4, 29.4, 29.3, 26.1, 26.1 (*C*H_2_), 22.7,22.7 (*C*H_2_CH_3_), 14.1 (*C*H_3_). HRMS (ESI, MeCN): calc. for [M+H] ^+^ (*m/z)*:1003.7022; Found: 1003.7020.

*Vin9-10: 4'-((4-(non-8-en-1-yloxy) benzoyl)oxy)-[1,1'-biphenyl]-4-yl3,4,5-tris(decyloxy)-benzoate, yield 69%, white solid.*

^1^H NMR (600 MHz, CDCl_3_) δ 8.17 (d, *J* = 8.8 Hz, 2H, Ar-*H*), 7.63 (dd, *J* = 8.5, 1.5 Hz, 4H, Ar-*H*), 7.43 (s, 2H, Ar-*H*), 7.28 (t, *J* = 8.5 Hz, 4H, Ar-*H*), 6.98 (d, *J* = 8.9 Hz, 2H, Ar-*H*), 5.82 (ddt, *J* = 16.9, 10.2, 6.7 Hz, 1H, -C*H*=), 5.01 (dd, *J* = 17.1, 1.8 Hz, 1H, =C*H_a_*H_b_), 4.95 (dd, *J* = 10.2, 0.9 Hz, 1H, =CH_a_*H_b_*), 4.28 - 3.83 (m, 8H, OC*H_2_*CH_2_), 2.06 (dd, *J* = 14.1, 6.9 Hz, 2H, C*H_2_*CH), 1.94 - 1.70 (m, 8H, OCH_2_C*H_2_*), 1.50 (dt, *J* = 15.2, 9.1 Hz, 10H, OCH_2_CH_2_C*H_2_*+CHCH_2_CH_2_C*H_2_*), 1.45 - 1.16 (m, 42H, C*H_2_*), 0.88 (td, *J* = 7.0, 4.1 Hz, 9H, C*H_3_*). ^13^C NMR (151 MHz, CDCl_3_) δ 165.1, 165.0 (*C*=O), 163.6, 153.0, 150.6, 150.5, 143.0 (*C_Ar_*-O), 139.1 (*C*H=), 138.2, 138.0(*C_Ar_*-*C_Ar_*), 132.3, 128.2, 128.2 (*C_Ar_*-H), 124.8, 123.9 (*C_Ar_*-COO-Ar) , 122.2, 122.1 (*C_Ar_*-H), 121.5 (*C_Ar_*-COO-Ar), 114.3 (*C*H_2_=), 114.3, 108.6 (*C_Ar_*-H), 73.6, 69.3, 68.3 (Ar-O*C*H_2_), 33.8 (*C*H_2_CH), 32.0, 31.9, 30.4, 29.7, 29.7, 29.6, 29.6, 29.4, 29.4, 29.3, 29.2, 29.1, 29.0, 28.8, 26.1, 26.1, 25.9 (*C*H_2_), 22.7, 22.7 (*C*H_2_CH_3_), 14.1 (*C*H_3_). HRMS (ESI, MeCN): calc. for [M+H] ^+^ (*m/z)*: 1003.7022; Found: 1003.7034.

*Vin9-12: 4'-((4-(non-8-en-1-yloxy) benzoyl) oxy)-[1,1'-biphenyl]-4-yl-3,4,5-tris-(dodecyloxy) benzoate, yield 72%, white solid.*

^1^H NMR (600 MHz, CDCl_3_) δ 8.17 (d, *J* = 8.5 Hz, 2H, Ar-*H*), 7.63 (d, *J* = 7.6 Hz, 4H, Ar-*H*), 7.43 (s, 2H, Ar-*H*), 7.33 - 7.17 (m, 4H, Ar-*H*), 6.98 (d, *J* = 8.5 Hz, 2H, Ar-*H*), 5.82 (dt, *J* = 16.7, 8.4 Hz, 1H, -C*H*=), 5.01 (d, *J* = 17.1 Hz, 1H, =C*H_a_*H_b_), 4.95 (d, *J* = 9.8 Hz, 1H, =CH_a_*H_b_*), 4.06 (d, *J* = 4.9 Hz, 8H, OC*H_2_*CH_2_), 2.06 (d, *J* = 6.6 Hz, 2H, C*H_2_*CH), 1.92 - 1.65 (m, 8H, OCH_2_C*H_2_*), 1.49 (s, 10H, OCH_2_CH_2_C*H_2_*+CHCH_2_CH_2_C*H_2_*), 1.43 - 1.21 (m, 54H, C*H_2_*), 0.87 (d, *J* = 6.9 Hz, 9H, C*H_3_*). ^13^C NMR (151 MHz, CDCl_3_) δ 165.1, 165.0 (*C*=O), 163.6, 153.0, 150.6, 150.5, 143.0 (*C_Ar_*-O), 139.1 (*C*H=), 138.2, 138.0(*C_Ar_*-*C_Ar_*), 132.3, 128.2, 128.2 (*C_Ar_*-H), 123.9 (*C_Ar_*-COO-Ar), 122.2, 122.1 (*C_Ar_*-H), 121.5 (*C_Ar_*-COO-Ar), 114.4 (*C*H_2_=), 114.3, 108.6(*C_Ar_*-H), 73.6, 69.3, 68.3(Ar-O*C*H_2_), 33.8 (*C*H_2_CH), 32.0, 31.9, 30.4, 29.8, 29.7, 29.7, 29.6, 29.6, 29.4, 29.4, 29.3, 29.2, 29.1, 29.0, 28.8, 26.1, 26.1, 25.9 (*C*H_2_), 22.7 (*C*H_2_CH_3_), 14.1 (*C*H_3_). HRMS (ESI, MeCN): calc. for [M+H] ^+^ (*m/z)*: 1087.7961; Found: 1087.7945.

*Vin7-10: 4'-((4-(hept-6-en-1-yloxy) benzoyl) oxy)-[1,1'-biphenyl]-4-yl3,4,5tris(decyloxy)benzoate, yield 71%, white solid.*

^1^H NMR (600 MHz, CDCl_3_) δ 8.17 (d, *J* = 8.7 Hz, 2H, Ar-*H*), 7.80 - 7.54 (m, 4H, Ar-*H*), 7.43 (s, 2H, Ar-*H*), 7.28 (t, *J* = 8.2 Hz, 4H, Ar-*H*), 6.98 (d, *J* = 8.54 Hz, 2H, Ar-*H*), 5.83 (ddt, *J* = 16.9, 10.2, 6.7 Hz, 1H, -C*H*=), 5.03 (dd, *J* = 17.1, 2.0 Hz, 1H, =C*H_a_*H_b_), 4.97 (d, *J* = 10.2 Hz, 1H, =CH_a_*H_b_*), 4.06 (td, *J* = 6.5, 2.7 Hz, 8H, OC*H_2_*CH_2_), 2.11 (q, *J* = 6.7 Hz, 2H, C*H_2_*CH), 1.85 (q, *J* = 7.0 Hz, 6H, OCH_2_C*H_2_*) , 1.77 (p, *J* = 6.9 Hz, 2H, OCH_2_C*H_2_*), 1.50 (tq, *J* = 10.1, 6.5, 5.3 Hz, 10H OCH_2_CH_2_C*H_2_*+CHCH_2_C*H_2_*), 1.41 – 1.22 (m, 36H, C*H_2_*), 0.88 (td, *J* = 6.8, 4.0 Hz, 9H, C*H_3_*). ^13^C NMR (151 MHz, CDCl_3_) δ 165.1, 165.0 (*C*=O), 163.6, 153.0, 150.6, 150.5, 143.0 (*C_Ar_*-O), 139.0 (*C*H=), 138.2, 138.0 (*C_Ar_*-*C_Ar_*), 132.4, 128.2, 128.2, 124.8 (*C_Ar_*-H), 123.9 (*C_Ar_*-COO-Ar), 122.2, 122.1 (*C_Ar_*-H), 121.5 (*C_Ar_*-COO-Ar), 114.6 (*C*H_2_=), 114.3, 108.6 (*C_Ar_*-H), 73.6, 69.3, 68.2 (Ar-O*C*H_2_), 33.7 (*C*H_2_CH), 32.0, 31.9, 30.4, 29.8, 29.7, 29.6, 29.6, 29.4, 29.4, 29.3, 29.0, 28.6, 26.1, 26.1, 25.5 (*C*H_2_), 22.7, 22.7 (*C*H_2_CH_3_), 14.1 (*C*H_3_).HRMS (APCI, MeCN): calc. for [M+H] ^+^ (*m/z)*: 975.6709; Found: 975.6664. Elemental analysis: calc. for C_63_H_90_O_8_: C 77.58%, H 9.30%, found: C 77.55%, H 9.59%.

*Vin7-12: 4'-((4-(hept-6-en-1-yloxy) benzoyl) oxy)-[1,1'-biphenyl]-4-yl3,4,5-tris(dodecyloxy)-benzoate, yield 76%, white solid.*

^1^H NMR (600 MHz, CDCl_3_) δ 8.17 (d, *J* = 7.7 Hz, 2H, Ar-*H*), 7.63 (d, *J* = 8.3 Hz, 4H, Ar-*H*), 7.43 (s, 2H, Ar-*H*), 7.28 (dd, *J* = 15.2, 7.2 Hz, 4H, Ar-*H*), 6.98 (d, *J* = 7.8 Hz, 2H, Ar-*H*), 5.83 (d, *J* = 7.0 Hz, 1H, -C*H*=), 5.03 (dd, *J* = 17.1, 1.6 Hz, 1H, =C*H_a_*H_b_), 4.97 (d, *J* = 10.2 Hz, 1H, =CH_a_*H_b_*), 4.06 (q, *J* = 6.2 Hz, 8H, OC*H_2_*CH_2_), 2.28 – 1.98 (m, 2H, C*H_2_*CH), 1.98 – 1.80 (m, 6H, OCH_2_C*H_2_*), 1.80 – 1.68 (m, 2H, , OCH_2_C*H_2_*), 1.50 (d, *J* = 5.6 Hz, 10H, OCH_2_CH_2_C*H_2_*+CHCH_2_C*H_2_*), 1.44 – 1.10 (m, 48H, C*H_2_*), 0.87 (d, *J* = 7.0 Hz, 9H, C*H_3_*). ^13^C NMR (151 MHz, CDCl_3_) δ 165.1, 165.0 (C=O), 163.5, 153.00, 150.6, 150.5, 143.0 (*C_Ar_*-O), 138.7 (*C*H=), 138.2, 138.0 (*C_Ar_*-*C_Ar_*), 132.3, 128.2, 128.2 (*C_Ar_*-H), 123.8 (*C_Ar_*-COO-Ar), 122.2, 122.1 (*C_Ar_*-H), 121.5 (*C_Ar_*-COO-Ar), 114.6 (*C*H_2_=), 114.3, 108.6 (*C_Ar_*-H), 73.6, 69.3, 68.2 (Ar-O*C*H_2_), 33.6 (*C*H_2_CH), 31.9, 31.9, 30.3, 29.8, 29.7, 29.7, 29.6, 29.6, 29.6, 29.4, 29.4, 29.3, 29.0, 28.6, 26.1, 26.1, 25.5 (*C*H_2_), 22.7 (*C*H_2_CH_3_), 14.1 (*C*H_3_). HRMS (APCI, MeCN): calc. for [M+H] ^+^ (*m/z)*: 1059.7647; Found: 1059.7629. Elemental analysis: calc. for C_69_H_102_O_8_: C 78.22%, H 9.70%, found: C 77.89%, H 9.73%.

*VinA9-7: 4'-((4-(non-8-en-1-yloxy) benzoyl) oxy)-[1,1'-biphenyl]-4-yl3,4,5-tris(he-ptyloxy)-benzoate，yield 70%, white solid.*

^1^H NMR (600 MHz, CDCl3) δ 8.17 (d, J = 8.6 Hz, 2H, Ar-H), 7.63 (dd, J = 8.5, 1.8 Hz, 4H, Ar-*H*), 7.43 (s, 2H, Ar-*H*), 7.28 (t, J = 8.1 Hz, 4H, Ar-*H*), 6.98 (d, J = 8.6 Hz, 2H, Ar-*H*), 5.82 (ddt, J = 16.9, 10.2, 6.6 Hz, 1H, -C*H*=), 5.01 (dd, J = 17.1, 2.0 Hz, 1H, =C*H*_a_H_b_), 4.95 (d, J = 10.1 Hz, 1H, =CH_a_*H_b_*), 4.06 (p, J = 6.2 Hz, 8H, OC*H_2_*CH_2_), 2.06 (q, J = 7.0 Hz, 2H, C*H_2_*CH), 1.84 (h, J = 7.1 Hz, 6H, OCH_2_C*H_2_*), 1.77 (p, J = 6.8 Hz, 2H, OCH_2_C*H_2_*), 1.53 - 1.45 (m, 8H, OCH_2_CH_2_C*H_2_*), 1.45 - 1.23 (m, 24H, C*H_2_*), 0.90 (td, J = 6.8, 2.7 Hz, 9H, C*H_3_*).^13^C NMR (151 MHz, CDCl_3_) δ 165.1, 165.0 (*C*=O), 163.6, 153.0, 150.6, 150.5, 143.0 (*C_Ar_*-O), 139.1 (*C*H=), 138.2, 138.0 (*C_Ar_*-*C_Ar_*), 132.3, 128.2, 128.2 (*C_Ar_*-H), 123.9 (*C_Ar_*-COO-Ar), 122.2, 122.1 (*C_Ar_*-H), 121.5 (Ar-*C*-COO-Ar), 114.3 (*C*H_2_=), 114.2, 108.6 (*C_Ar_*-H), 73.6, 69.3, 68.3(Ar-O-*C*H_2_), 33.8 (*C*H_2_CH), 31.9, 31.8, 30.4, 29.3, 29.2, 29.2, 29.1, 29.0, 28.9, 26.1, 26.0, 26.0 (*C*H_2_), 22.7, 22.6 (*C*H_2_CH_3_), 14.1 (*C*H_3_). HRMS (APCI, MeCN): calc. for [M+H] ^+^ (*m/z)*: 877.5613; Found: 877.5592. Elemental analysis: calc. for C_56_H_76_O_8_: C 76.68%, H 8.73%, found: C 76.58%, H 8.59%.

*VinA9-7F: 4'-((3-fluoro-4-(non-8-en-1-yloxy) benzoyl) oxy)-[1,1'-biphenyl]-4-yl 3,4,5-tris(heptyloxy)benzoate, yield 65%, white solid.*

^1^H NMR (600 MHz, CDCl_3_) δ 8.15 - 7.82 (m, 2H, *p*F-Ar-*H*, *o*F-Ar-*H*), 7.80 - 7.55 (m, 4H Ar-*H*), 7.43 (d, *J* = 2.8 Hz, 2H, Ar-*H*), 7.32 - 7.26 (m, 4H, Ar-*H*), 7.04 (td, *J* = 8.3, 2.9 Hz, 1H, *m*F-Ar-*H*), 5.88 - 5.76 (m, 1H, C*H*=), 5.09 - 4.98 (m, 1H, =C*H_a_*H_b_), 4.97 - 4.82 (m, 1H, =CH_a_*H_b_*), 4.13 (td, *J* = 6.5, 3.0 Hz, 2H, *o*F-Ar-O-C*H_2_*), 4.10 – 3.96 (m, 6H, OC*H_2_*CH_2_), 2.15 – 1.98 (m, 2H, C*H_2_*CH), 1.94 – 1.69 (m, 8H, OCH_2_C*H_2_*), 1.50 (d, *J* = 2.1 Hz, 8H, OCH_2_CH_2_C*H_2_*), 1.45 – 1.22 (m, 24H, C*H_2_*), 0.90 (dt, *J* = 9.7, 3.4 Hz, 9H, C*H_3_*). ^13^C NMR (151 MHz, CDCl_3_) δ 165.1, 164.2 (*C*=O), 153.0, 152.7 (*C_Ar_*-H) , 152.0, 152.0 (*C_Ar_*-O), 151.1 (*C_Ar_* -F), 150.6, 150.4, 143.1 (*C_Ar_*-H), 139.1 (*C*H=), 138.2, 138.1 (*C_Ar_*-*C_Ar_*), 128.2, 127.4, 127.4 (*C_Ar_*-H), 123.8 (*C_Ar_*-COO-Ar), 122.2, 122.1 (C-Ar), 121.8, 121.8 (*C_Ar_*-COO-Ar), 117.9 (*o*F-*C_Ar_*), 114.3 (*C*H_2_=), 113.4 (*m*F-*C_Ar_*-H), 108.6 (*C_Ar_*-H), 73.6, 69.4, 69.3 (Ar-O-*C*H_2_), 33.8 (*C*H_2_CH), 31.9, 31.8, 30.4, 29.7, 29.3, 29.2, 29.2, 29.1, 29.0, 28.8, 26.1, 26.0, 25.8 (*C*H_2_), 22.7, 22.6 (*C*H_2_CH_3_), 14.1 (*C*H_3_). HRMS (APCI, MeCN): calc. for [M+H] ^+^ (*m/z)*: 895.5519; Found: 895.5516. Elemental analysis: calc. for C_56_H_75_FO_8_: C 75.14%, H 8.44%, found: C 74.98%, H 8.57%.

*VinA9-7NO2: 4'-((3-nitro-4-(non-8-en-1-yloxy) benzoyl)oxy)-[1,1'-biphenyl]-4-yl 3,4,5-tris(heptyloxy)benzoate, yield 42%m white solid.*

^1^H NMR (600 MHz, CDCl3) δ 8.68 (d, *J* = 2.1 Hz, 1H, oNO2-Ar-*H*), 8.36 (dd, *J* = 8.8, 2.1 Hz, 1H, pNO2-Ar-*H*), 7.65 (t, *J* = 8.0 Hz, 4H, Ar-*H*), 7.43 (s, 2H, Ar-*H*), 7.29 (t, *J* = 7.9 Hz, 4H, -COO-Ar-*H*), 7.19 (d, *J* = 8.9 Hz, 1H, mNO2-Ar-*H*), 5.91 - 5.71 (m, 1H, C*H*=), 5.01 (d, *J* = 17.1 Hz, 1H, =C*H_a_*H_b_), 4.95 (d, *J* = 10.2 Hz, 1H, =CH_a_*H_b_*), 4.22 (t, *J* = 6.4 Hz, 2H, *o*NO2-Ar-OC*H_2_*CH_2_ ), 4.07 (dd, *J* = 11.8, 6.3 Hz, 6H, OC*H_2_*CH_2_), 2.06 (q, *J* = 7.0 Hz, 2H, C*H_2_*CH), 1.83 (tdd, *J* = 42.3, 14.7, 7.0 Hz, 8H, OCH_2_C*H_2_*), 1.52 - 1.29 (m, 32H, C*H_2_*), 0.92 - 0.87 (m, 9H, C*H_3_*). ^13^C NMR (151 MHz, CDCl_3_) δ 165.1, 163.2 (C=O), 156.2, 153.0, 150.6, 150.1, 143.07 (*C_Ar_*-O), 139.6 (*C_Ar_*-NO_2_), 139.1 (*C*H), 138.4, 138.0 (*C_Ar_*-*C_Ar_*), 135.8 (*p*NO_2_-*C_Ar_*-O), 128.3, 128.2, 127.8 (*C_Ar_*-H), 123.8 (*C_Ar_*-COO-Ar), 122.2, 121.9 (*C_Ar_*-H), 121.4 (*C_Ar_*-COO-Ar), 114.3 (*C*H_2_=), 114.0 (*m*NO_2_-*C_Ar_*-O), 108.6 (*C_Ar_*-H), 73.6, 70.2, 69.3 (Ar-O-*C*H_2_), 33.8 (*C*H_2_CH), 31.9, 31.8, 30.4, 29.3, 29.2, 29.1, 28.9, 28.8, 28.8, 26.1, 26.0, 25.8 (*C*H_2_), 22.7, 22.6 (*C*H_2_CH_3_), 14.1 (*C*H_3_). HRMS (APCI, MeCN): calc. for [M+H] ^+^ (*m/z)*: 922.5464; Found: 922.5459. Elemental analysis: calc. for C_56_H_75_FO_8_: C 72.93%, H 8.20%, N 1.52%, found: C 73.22%, H 8.58%, N 1.58%.

*VinA9-9:* *4'-((4-(non-8-en-1-yloxy) benzoyl) oxy)-[1,1'-biphenyl]-4-yl 3,4,5-tris(nonyloxy)benzoate, yield 74%, white solid.*

^1^H NMR (600 MHz, CDCl3) δ 8.17 (d, *J* = 8.8 Hz, 2H, Ar-*H*), 7.63 (dd, *J* = 8.3, 1.3 Hz, 4H, Ar-*H*), 7.43 (s, 2H, Ar-*H*), 7.28 (dd, *J* = 15.3, 6.9 Hz, 4H, Ar-*H*), 6.98 (d, *J* = 8.8 Hz, 2H, Ar-*H*), 5.82 (ddt, *J* = 16.9, 10.2, 6.7 Hz, 1H, -C*H*=), 5.06 - 4.97 (m, 1H, =C*H_a_*H_b_), 4.95 (d, J = 10.1 Hz, 1H, =C*H_a_*H_b_), 5.24 - 4.69 (m, 2H), 4.27 - 3.83 (m, 8H, OC*H_2_*CH_2_), 2.06 (q, *J* = 7.0 Hz, 2H, C*H_2_*CH), 1.97 - 1.64 (m, 8H, OCH_2_C*H_2_*), 1.49 (dd, *J* = 14.2, 7.1 Hz, 8H, OCH_2_CH_2_C*H_2_*), 1.45 - 1.14 (m, 36H, C*H_2_*), 0.89 (dd, *J* = 11.2, 6.8 Hz, 9H, C*H_3_*). ^13^C NMR (151 MHz, CDCl3) δ 165.1, 165.0 (*C*=O), 163.6, 153.0, 150.6, 150.5, 143.1 (*C_Ar_*-O), 139.1 (*C*H=), 138.3, 138.1 (*C_Ar_*-*C_Ar_*), 138.1, 132.4, 128.2, 128.2 (*C_Ar_*-H), , 123.9 (*C_Ar_*-COO-Ar), 122.2, 122.1 (*C_Ar_*-H), 121.5 (Ar-*C*-COO-Ar), 114.3 (*C*H_2_=), 114.3, 108.6 (*C_Ar_*-H), 73.6, 69.3, 68.3 (Ar-O-*C*H_2_), 33.8 (*C*H_2_CH), 32.0, 31.9, 31.5, 30.4, 29.7, 29.6, 29.6, 29.4, 29.4, 29.3, 29.2, 29.1, 29.0, 28.9, 26.1, 26.1, 26.0 (*C*H_2_), 22.7, 22.7 (*C*H_2_CH_3_), 14.2 (*C*H_3_). HRMS (APCI, MeCN): calc. for [M+H] ^+^ (*m/z)*: 961.6552; Found: 961.6556. Elemental analysis: calc. for C_62_H_88_O_8_: C 77.46%, H 9.23%, found: 77.43C %, H 9.24%.

*Synthesis of compounds Dim-n*

According to procedure *v*: To a solution of Vin *m-n* (2.2 eq.) and 1,1,3,3-tetramethyldisiloxane (1 eq.) in dry toluene (10 ml) was added 40ppm Karstedt’s catalyst dissolved in xylene. The mixture was gently aerated for 30 minutes and the reaction was carrying on at r.t. for 10 hours. The solution was concentrated under reduced pressure and the crude product was purified by column chromatography, the ratio of solvent PE/DCM is 1/2 for TLC, R_f_ is about 0.25, yield a white solid.

*Di3-10: ((4,4'-(((1,1,3,3-tetramethyldisiloxane-1,3-diyl) bis(propane-3,1-diyl))bis(oxy))bis(-benzoyl))bis(oxy))bis([1,1'-biphenyl]-4',4-diyl)bis(3,4,5-tris(dodecyloxy)-benzoate), yield 49%, white solid.*

^1^H NMR (600 MHz, CDCl_3_) δ 8.17 (d, *J* = 8.7 Hz, 4H, Ar-*H*), 7.63 (dd, *J* = 8.5, 1.9 Hz, 8H, Ar-*H*), 7.43 (s, 4H, Ar-*H*), 7.34 - 7.26 (m, 10H, Ar-*H* and C*H*Cl_3_), 6.99 (d, *J* = 8.8 Hz, 4H, Ar-*H*), 4.21 - 3.89 (m, 16H, OC*H_2_*CH_2_), 1.85 (ddd, *J* = 24.9, 14.0, 7.4 Hz, 12H, OCH_2_C*H_2_*), 1.76 (dd, *J* = 14.7, 7.0 Hz, 4H, , OCH_2_C*H_2_*CH_2_Si), 1.53 - 1.43 (m, 12H, OCH_2_CH_2_C*H_2_*), 1.42 - 1.19 (m, 72H, C*H_2_*), 0.89 (dt, *J* = 11.6, 5.8 Hz, 18H, C*H_3_*), 0.74 - 0.59 (m, 4H, SiC*H_2_*), 0.19 - 0.08 (m, 12H, SiC*H_3_*). ^13^C NMR (151 MHz, CDCl_3_) δ 165.1, 165.0 (*C*=O), 163.5, 153.0, 150.6, 150.5, 143.0 (*C_Ar_*-O), 138.2, 138.0 (*C_Ar_*-*C_Ar_*), 132.4, 128.2, 128.2 (*C_Ar_*-H), 123.9 (*C_Ar_*-COO-Ar), 122.2, 122.1 (*C_Ar_*-H), 121.5 (*C_Ar_*-COO-Ar), 114.3, 108.6 (*C_Ar_*-H), 73.6, 70.7, 69.3 (Ar-O*C*H_2_), 32.0, 31.9, 30.4, 29.8, 29.7, 29.7, 29.6, 29.6, 29.4, 29.4, 29.3, 26.1, 26.1, 23.1, 22.7, 22.7 (*C*H_2_), 14.3, 14.1 (*C*H_3_), 0.4 (Si*C*H_3_). HRMS (APCI, MeCN): calc. for [M+H] ^+^ (*m/z)*: 1972.2675; Found: 1972.2618. Elemental analysis: calc. for C_122_H_178_O_17_Si_2_: C 74.27%, H 9.09%, found: C 74.20%, H 9.23%.

*Di3-12: ((4,4'-(((1,1,3,3-tetramethyldisiloxane-1,3-diyl) bis(propane-3,1-diyl)) bis-(oxy)) bis(benzoyl)) bis(oxy)) bis([1,1'-biphenyl]-4',4-diyl) bis(3,4,5-tris(dodecyl-loxy) benzoate), yield 52%, white solid.*

^1^H NMR (600 MHz, CDCl_3_) δ 8.17 (d, *J* = 8.6 Hz, 4H, Ar-*H*), 7.63 (d, *J* = 6.8 Hz, 8H, Ar-*H*), 7.42 (s, 4H, Ar-*H*), 7.36 - 7.16 (m, 16H, Ar-*H*+C*H*Cl_3_), 6.99 (d, *J* = 8.8 Hz, 4H, Ar-*H*), 4.32 - 3.72 (m, 16H, OC*H_2_*CH_2_), 2.05 - 1.69 (m, 16H, OCH_2_C*H_2_*), 1.48 (dd, *J* = 14.7, 8.0 Hz, 12H, OCH_2_CH_2_C*H_2_*), 1.43 - 1.17 (m, 96H, C*H_2_*), 0.88 (td, *J* = 6.9, 3.7 Hz, 18H, C*H_3_*), 0.74 - 0.56 (m, 4H, SiC*H_2_*), 0.24 - 0.09 (m, 12H, SiC*H_3_*). ^13^C NMR (151 MHz, CDCl_3_) δ 165.1, 165.0 (*C*=O), 163.5, 153.0, 150.6, 150.5, 143.0 (*C_Ar_*-O), 138.2, 138.0 (*C_Ar_*-*C_Ar_*), 132.4, 128.2, 128.2(*C_Ar_*-H), 123.9 (*C_Ar_*-COO-Ar), 122.2, 122.1 (*C_Ar_*-H), 121.5 (*C_Ar_*-COO-Ar), 114.3, 108.6 (*C_Ar_*-H), 73.6, 70.7, 69.3 (Ar-O*C*H_2_), 32.0, 30.4, 29.8, 29.7, 29.7, 29.7, 29.6, 29.4, 29.4, 29.3, 26.1, 26.1, 23.1, 22.7 (*C*H_2_), 14.3, 14.1 (*C*H_3_), 0.4 (Si*C*H_3_). HRMS (APCI, MeCN and THF): calc. for [M+H] ^+^ (*m/z)*: 2140.4553; Found: 2140.4452. Elemental analysis: calc. for C_134_H_202_O_17_Si_2_: C 75.17%, H 9.51%, found: C 74.95%, H 9.71%.

*Di7-12: ((4,4'-(((1,1,3,3-tetramethyldisiloxane-1,3-diyl) bis(heptane-7,1-diyl))bis-(oxy))bis(benzoyl))bis(oxy))bis([1,1'-biphenyl]-4',4-diyl)bis(3,4,5-tris(dodecylo-xy)benzoate), yield 51%, white solid.*

^1^H NMR (600 MHz, CDCl_3_) δ 8.16 (d, *J* = 7.9 Hz, 4H, Ar-*H*), 7.63 (d, *J* = 7.8 Hz, 8H, Ar-*H*), 7.43 (s, 4H, Ar-*H*), 7.36 - 7.16 (m, 10H, Ar-*H* and C*H*Cl_3_), 6.98 (d, *J* = 7.9 Hz, 4H, Ar-*H*), 4.06 (s, 16H, OC*H_2_*CH_2_), 2.04 - 1.68 (m, 16H, OCH_2_C*H_2_*), 1.49 (s, 16H, OCH_2_CH_2_C*H_2_*), 1.31 (d, *J* = 58.5 Hz, 108H, C*H_2_*), 0.88 (s, 18H, C*H_3_*), 0.52 (s, 4H, SiC*H_2_*), 0.06 (d, *J* = 14.9 Hz, 12H, SiC*H_3_*). ^13^C NMR (151 MHz, CDCl_3_) δ 165.1, 165.0(C=O), 163.6, 153.0, 150.6, 150.5, 143.0 (*C_Ar_*-O), 138.2, 138.0 (*C_Ar_*-*C_Ar_*), 132.3, 128.2, 128.2 (*C_Ar_*-H), 123.9 (*C_Ar_*-COO-Ar), 122.2, 122.1 (*C_Ar_*-H), 121.5 (*C_Ar_*-COO-Ar), 114.3, 108.6 (*C_Ar_*-H), 73.6, 69.3, 68.3 (Ar-O*C*H_2_), 33.3, 32.0, 32.0, 30.4, 29.8, 29.7, 29.7, 29.7, 29.6, 29.4, 29.4, 29.3, 29.2, 29.1, 26.1, 26.0, 23.3, 22.7(*C*H_2_), 18.4(SiCH_2_*C*H_2_), 14.1 (C*H_3_*), 0.4 (SiC*H_3_*). Elemental analysis: calc. for C_142_H_218_O_17_Si_2_: C 75.69%, H 9.75%, found: C 75.53%, H 9.93%. Η-Η COSY, HMBC and HSQC have been analyzed in Table 1.

*Synthesis of compounds Sim-n*

According to procedure *v*: A mixture of compound VinA *m-n* (1.1 eq.), polymethylhydrosiloxane (PMHS) (1 eq.), 5 mL of dry toluene, and several drops of Karstedt’s catalyst were added in a 25 mL round-bottom flask. The reaction was purged with nitrogen and heated for 48 hours at 60 ℃. The product was purified by Al_2_O_3_ twice and precipitated by methyl alcohol and acetone three times to yield the white solid.

*Si9-10**: yield 52%, white solid.*

Mn,_GPC_=37300 g/mol, Mw=64800 g/mol, PDI=1.74.

^1^H NMR (600 MHz, CDCl_3_) δ 8.10 (s, 2H, Ar-*H*), 7.54 (s, 4H, Ar-*H*), 7.38 (s, 2H, Ar-*H*), 7.21 (s, 4H, Ar-*H*), 6.91 (s, 2H, Ar-*H*), 4.75 (s, 0.05H, Si-*H*), 4.01 (d, *J* = 15.9 Hz, 8H, OC*H_2_*CH_2_), 2.00 - 1.64 (m, 8H, OCH_2_C*H_2_*), 1.52 - 1.09 (m, 66.5H, C*H_2_*), 1.02 - 0.68 (m, 9H, C*H_3_*), 0.56 (s, 2H, SiC*H_2_*), 0.09 (d, *J* = 21.3 Hz, 4H, SiC*H_3_*). *p* was calculated by the H in position 8.10 and 4.75. ^13^C NMR (151 MHz, CDCl_3_) δ 165.0 (*C*=O), 163.4, 152.97, 150.52, 143.08 (*C_Ar_*-O), 138.0 (*C_Ar_*-*C_Ar_*), 132.3, 128.10, 123.82, 122.2, 114.2, 108.5 (*C_Ar_*-H), 73.6, 69.2, 68.3 (O*C*H_2_), 33.6 (SiCH*_2_*CH_2_*C*H_2_), 32.0, 31.9, 30.4, 29.8, 29.7, 29.6, 29.6, 29.4, 29.4, 29.3 (*C*H_2_), 26.1 (Si*C*H_2_CH_2_), 22.7 (*C*H_2_), 17.8 (SiCH_2_*C*H_2_), 14.1 (*C*H_3_), 1.0 (Si*C*H_3_).

*Si9-12: yield 56%, white solid.*

Mn, _GPC_=46700 g/mol, Mw=78300 g/mol, PDI=1.66.

^1^H NMR (600 MHz, CDCl_3_) δ 8.10 (s, 2H, Ar-*H*), 7.54 (s, 4H, Ar-*H*), 7.38 (s, 2H, Ar-*H*), 7.21 (s, 4H, Ar-*H*), 6.91 (s, 2H, Ar-*H*), 4.75 (s, 0.05H, Si-*H*), 4.01 (d, *J* = 15.9 Hz, 8H, OC*H_2_*CH_2_), 2.02 - 1.66 (m, 8H, OCH_2_C*H_2_*), 1.52 - 1.06 (m, 66.7H, C*H_2_*), 1.04 - 0.71 (m, 9H, C*H_3_*), 0.56 (s, 2H, SiC*H_2_*), 0.09 (d, *J* = 21.3 Hz, 4H, SiC*H_3_*). *p* was calculated by the H in position 8.10 and 4.75. ^13^C NMR (151 MHz, CDCl_3_) δ 165.1 (*C*=O), 163.5, 153.0, 150.5, 143.0 (*C_Ar_*-O), 138.0 (*C_Ar_*-*C_Ar_*), 132.3, 128.1, 123.9, 122.1, 114.2, 108.6 (*C_Ar_*-H), 73.6, 69.2, 68.3 (O*C*H_2_), 33.6 (SiCH*_2_*CH_2_*C*H_2_), 32.0, 31.9, 30.4, 29.8, 29.7, 29.6, 29.6, 29.4, 29.4, 29.3 (*C*H_2_), 26.1 (Si*C*H_2_CH_2_), 22.7 (*C*H_2_), 17.8 (SiCH_2_*C*H_2_), 14.1 (*C*H_3_), 1.0 (Si*C*H_3_).

**9.2 NMR and MS spectra**





**Figure S15. a.** ^1^H-NMR (600 MHz) and **b.** ^13^C-NMR (151 MHz) spectrum of Vin7-10 in CDCl_3_, 298K.

**
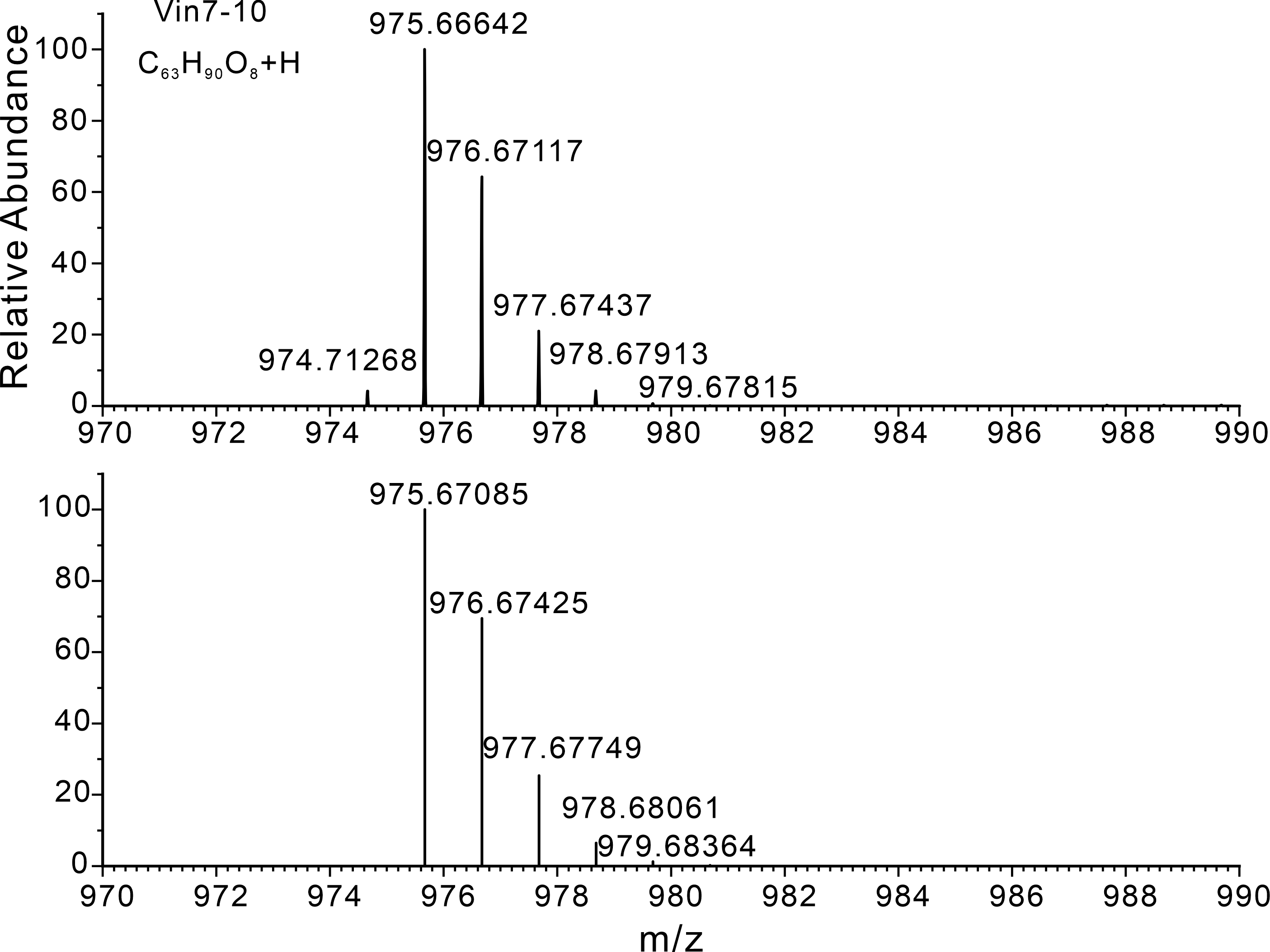
**

**Figure S16.** Measured (upper) and Simulated (bottom) HRMS of Vin7-10 (APCI, MeCN).





**Figure S17. a.** ^1^H-NMR (600 MHz) and **b.** ^13^C-NMR (151 MHz) spectrum of Vin7-12 in CDCl_3_, 298K.





**Figure S18.** Measured (upper) and Simulated (bottom) HRMS of Vin7-12 (APCI, MeCN).





**Figure S19. a.** ^1^H-NMR (600 MHz) and **b.** ^13^C-NMR (151 MHz) spectrum of Vin9-7 in CDCl_3_, 298K.





**Figure S20.** Measured (upper) and Simulated (bottom) HRMS of Vin9-7 (APCI, MeCN).





**Figure S21. a.** ^1^H-NMR (600 MHz) and **b.** ^13^C-NMR (151 MHz) spectrum of Vin9-7F in CDCl_3_, 298K.


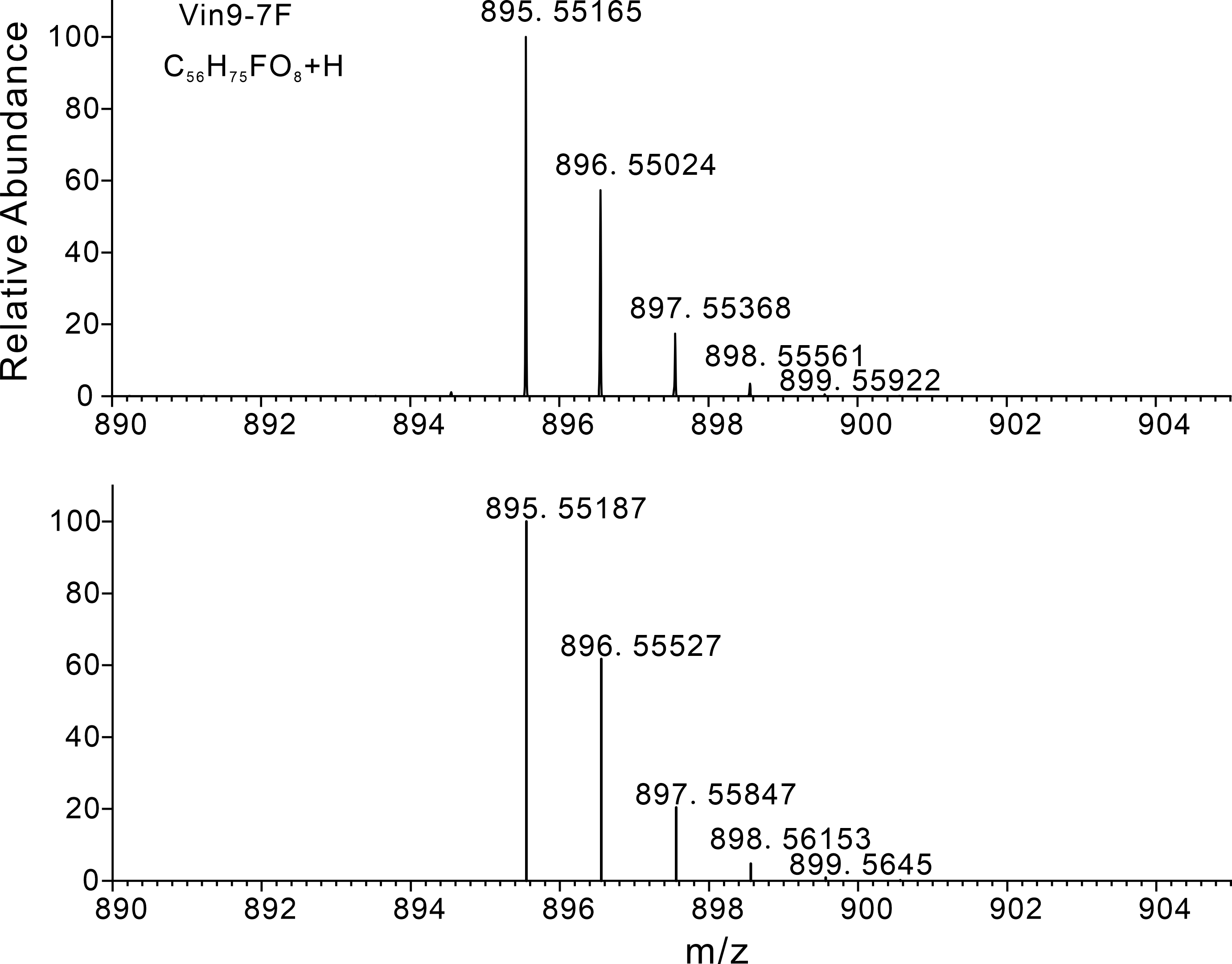


**Figure S22.** Measured (upper) and Simulated (bottom) HRMS of Vin9-7F (APCI, MeCN).


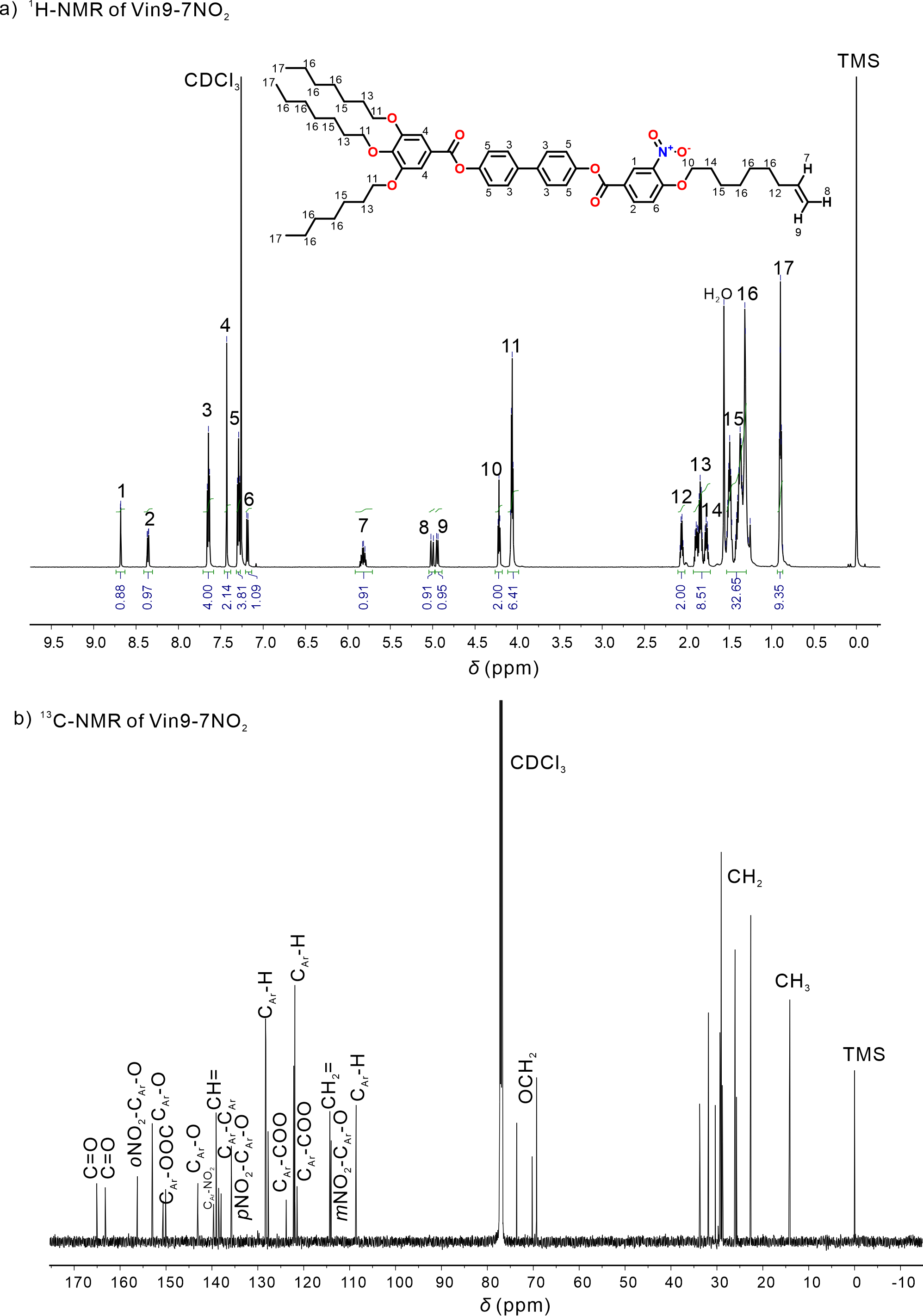


**Figure S23. a.** ^1^H-NMR (600 MHz) and **b.** ^13^C-NMR (151 MHz) spectrum of Vin9-7NO_2_ in CDCl_3_, 298K.





**Figure S24.** Measured (upper) and Simulated (bottom) HRMS of Vin9-7NO_2_ (APCI, MeCN).





**Figure S25. a.** ^1^H-NMR (600 MHz) and **b.** ^13^C-NMR (151 MHz) spectrum of Vin9-9 in CDCl_3_, 298K.





**Figure S26.** Measured (upper) and Simulated (bottom) HRMS of Vin9-9 (APCI, MeCN).





**Figure S27. a.** ^1^H-NMR (600 MHz) and **b.** ^13^C-NMR (151 MHz) spectrum of Di3-10 in CDCl_3_, 298K.





**Figure S28.** Measured (upper) and Simulated (bottom) HRMS of Di3-10 (APCI, MeCN and THF).





**Figure S29. a.** ^1^H-NMR (600 MHz) and **b.** ^13^C-NMR (151 MHz) spectrum of Di3-12 in CDCl_3_, 298K.





**Figure S30.** Measured (upper) and Simulated (bottom) HRMS of Di3-12 (APCI, MeCN and THF).





**Figure S31. a.** ^1^H-NMR (600 MHz) and **b.** ^13^C-NMR (151 MHz) spectrum of Di7-12 in CDCl_3_, 298K.





**Figure S32.** H-H COSY spectrum of Di7-12 (150 MHz, CDCl_3_, 298K)





**Figure S33.** HMBC spectrum of Di7-12(150 MHz, CDCl_3_, 298K)





**Figure S34.** HSQC spectrum of Di7-12 (150 MHz, CDCl_3_, 298K)





**Figure S35. a.** ^1^H-NMR (600 MHz) and **b.** ^13^C-NMR (151 MHz) spectrum of PMHS in CDCl_3_. There was no internal standard substance TMS in CDCl_3_, 298K.





**Figure S36. a.** ^1^H-NMR (600 MHz) and **b.** ^13^C-NMR (151 MHz) spectrum of Si9-10 in CDCl_3_, 298K.





**Figure S37. a.** ^1^H-NMR (600 MHz) and **b.** ^13^C-NMR (151 MHz) spectrum of Si9-12 in CDCl_3_, 298K.

**Table S11.** Analysis of ^1^H-NMR (600 MHz), ^13^C-NMR (151 MHz), ^1^H-^1^H COSY, HSQC and HMBC for compound Di7-12. Solvent is CDCl_3_, the serial numbers of carbons and hydrogens are shown below.

| **Position** | **^1^H-NMR** | **^13^C-NMR** | **COSY H-H** | **HSQC** | **HMBC** |
| --- | --- | --- | --- | --- | --- |
| 1, 13, 25 | 0.88, m, 9H | 14.2 | 0.88 -[1.29-1.3.1]  H1-H2; H13-H14,  H25-H24 | 0.88-14.3 | H1,13,25 <> C3, 15, 23 = 32.1  > C2, 14, 24 = 22.8 |
| 2-9,  14-21,  26-33 | 1.2-1.4, 54H (48 H from pos. 66-68) | 22.7, 23.3, 29.3-29.6, 32.1, 33.4 |  | 1.36 – 23,3, 29.5, 29.4, 33.4; 1.29 – 22.7; 1.26- 29.6, 32.1 | 1.28 <> C1,13,25 = 14.2  1.25 – 22.8  1.26 – 29.7  1.29 – 32.0  1.36 – 29.7 |
| 10, 22, 34 | 1.49, m, 8H (2H from pos. 65) | 26.1, 26.2 | 1.49- 26.2 | 1.49-26.1  1.49-26.2 | H10 <> C8 = 29.5  H22 <> C20 = 29.5  H34 <> C32 = 29.5 |
| 11, 35 | 1.84, m, 6H (2H from pos. 64) | 29.2 | 1.84 – 4.05  H11- H12; H35 – H36  1.84 – 1.49  H11 – H10; H35 – H34 | 1.84-29.3 | H11, 35 <> C12,36 = 69.4  > C9, 33 = 29.3  > C10, 34 = 26.1 |
| 23 | 1.77, m, 2H | 30.5 | 1.77- 4.04  H23 – H24  1.77 – 1.49  H23-H22 | 1.77-30.5 | H23 <> C24 = 73.7  > C21 = 29.6  > C22 = 26.2 |
| 12, 36 | 4.05, m, 6H (2H from pos. 63) | 69.4 | 4.05 – 1.84  H12- H11  H36 – H35 | 4.05 - 69.4 | H12,36 <> C37,38 = 153.1  > C11,35 = 29.2  > C10,34 = 26.1 |
| 24 | 4.06, m, 2H | 73.7 | 4.06 – 1.77  H24 – H23 | 4.06 - 73.64 | H24 <> C39 = 143.2  > C23 = 30.5  > C22 = 26.2 |
| 37, 38 | - | 153.1 |  |  |  |
| 39 | - | 143.2 |  |  |  |
| 40, 41 | 7.43, s, 2H | 108.7 |  | 7.43 – 108.7 | H40, 41<> C43 = 165.2  > C37, 38 = 153.1  > C39 = 143.2  > C42 = 124.0  > C40, 41 = 108.7 |
| 42 | - | 124.0 |  |  |  |
| 43 | - | 165.2 |  |  |  |
| 44 | - | 150.7 |  |  |  |
| 45, 46 | 7.28, m, 4H (2H from pos. 53 and 54) | 122.3 | 7.28-7.63  H45,46 – H47,48 | 7.28 – 122.3 | H45, 46<> C44 = 150.7  > C49 = 138.3  > C45, 46 = 122.3 |
| 47, 48 | 7.63, dd, 4H (2H from pos. 51 and 52) | 128.3 | 7.63-7.28  H47,48 – H45,46 | 7.63 – 128.3 | H47, 48<> C44 = 150.7  > C50 = 138.2  > C47, 48 = 128.3 |
| 49 | - | 138.3 |  |  |  |
| 50 | - | 138.2 |  |  |  |
| 51,52 | 7.63, dd, 4H (2H from pos. 47 and 48) | 128.3 | 7.63-7.28  H51,52 – H53,54 | 7.63 – 128.3 | H51, 52<> C55 = 150.7  > C49 = 138.3  > C51,52 = 128.3 |
| 53, 54 | 7.28, m, 4H (2H from pos. 45 and 46) | 122.3 | 7.28 -7.63  H53, 54 – H51,52 | 7.28 – 122.3 | H53, 54< > C55 = 150.7  > C50 = 138.2  > C53, 54 = 122.3 |
| 55 | - | 150.7 |  |  |  |
| 56 | - | 165.1 |  |  |  |
| 57 | - | 121.6 |  |  |  |
| 58, 59 | 8.16, d, 2H | 132.5 | 8.16 - 6.98  H58,59-H60,61 | 8.16 – 132.5 | H58,59<> C56 = 165.1  > C62 = 163.7  > C58, 59 = 132.5 |
| 60, 61 | 6.98, d, 2H | 114.5 | 6.98 - 8.16  H60,61 – H58, 59 | 6.98 – 114.5 | H60,61 <> C62 = 163.7  > C57 = 121.6  > C60, 61 = 1114.4 |
| 62 | - | 163.7 |  |  |  |
| 63 | 4.05, m, 6H (4H from pos. 12 and 36) | 68.5 | 4.05 – 1.84  H63 – H64 | 4.05 – 68.5 | H63 <> C62 = 163.7  > C64 = 29.2  > C65 = 26.1 |
| 64 | 1.84, m, 6H (4H from pos. 11 and 35) | 29.2 | 1.84 – 4.05  H64 – H63  1.84- 1.49  H64-H65 | 1.84 – 29.3 | H64 <> C63 = 68.5  > C66 = 29.3  > C65 = 26.1 |
| 65 | 1.49, m, 8H (6H from pos. 10, 22, 34) | 26.1 |  | 1.49 – 26.1 | H65 <> C67 = 29.5 |
| 66-67 | 1.20-1.42 overlap, 54 H (50 H from pos.: 2-9, 14-21, 26-33, 68) | 29.4-29.7 |  |  |  |
| 68 | 1.36 overlap,54H (52 H from pos.: 2-9, 14-21, 26-33, 65-67) | 33.4 | 1.36 – 0.52  H68 – H69 | 1.36 – 33.4 | H68 <> C66 = 29.7 |
| 69 | 0.52, t, 2H | 18.5 | 0.52 – 1.36  H69 – H68 | 0.52 – 18.5 | H69 <> C68 = 33.4  > C67 = 23.4 |
| 70, 71 | 0.05, s, 6H | 0.5 | - | 0.05 – 0.5 | H70, 71 <> C69 = 18.5  > C70, 71 = 0.5 |

# Additional imaging

Attempts have been made to capture images of Iso* melt. Two methods were tried, freeze-fracture TEM and AFM of material quenched from the Iso* phase. Compound Vin9-7F at 50°C was chosen as the formation of the LC phase can be easily suppressed by quenching. However, to suppress crystallization this vinyl-groups containing monomer was UV irradiated at 50°C, i.e. at the temperature where the metastable Iso* has a long lifetime. The sample was cooled from the Iso melt to 50°C and crosslinked by UV irradiation. Irradiation time was adjusted to be long enough to eliminate the crystallization exotherm in the subsequent DSC test.

The AFM and FFTEM methods used are described in Section 8 above, and the representative images are shown below. We consider the images featureless.


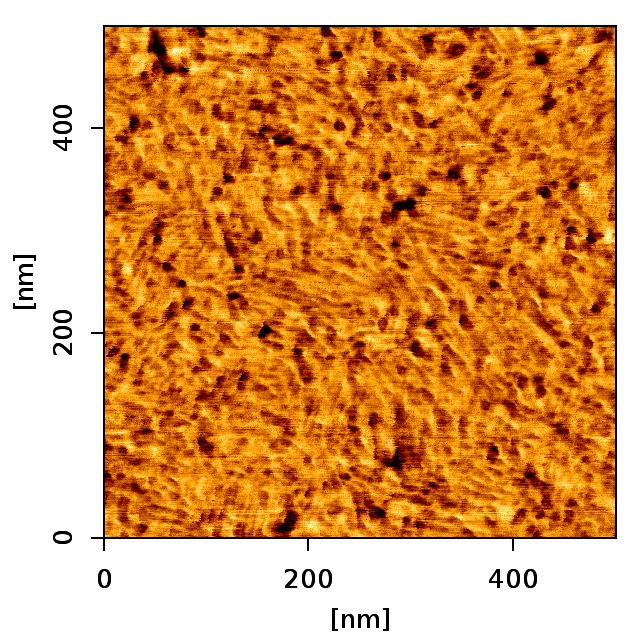

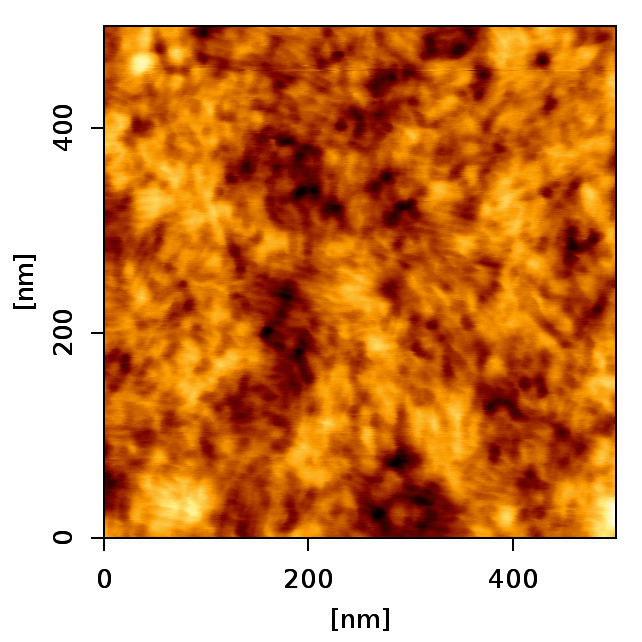


**Figure S38**. AFM images of Vin9-7F crosslinked in the Iso* phase 50°C and quenched to room temperature. Left: height image, right: phase image. The faint horizontal stripes are scanning artefacts.


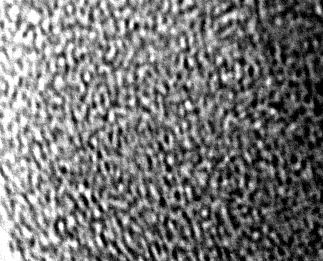


**Figure S39. Freez-francture TEM of Vin9-7F crosslinked in the Iso* phase at 50°C and quenched to room temperature.**

1. References

1. S. N. Sebastian, M. R. de la Fuente, D. O. Lopez, M. A. Perez-Jubindo, J. Salud, S. Diez-Berart, M. B. Ros, *J. Phys. Chem. B* **2011**, *115*, 9766-9775. [↑](#endnote-ref-1)
2. S. G. S. Attard, C. T. Imrie, F. E. Karasz, *Chem. Mater.* **1992**, *4*, 1246. [↑](#endnote-ref-2)
3. S. E. M. Barrall, II, R. S. Porter, J. F. Johnson, *J. Phys. Chem.* **1964**, *68*, 2810. [↑](#endnote-ref-3)
4. S. V. Percec, J. Heck, G. Ungar, *Macromolecules* **1991**, *24*, 4957-4962. [↑](#endnote-ref-4)
5. S. X. B. Zeng, L. Cseh, G. H. Mehl, G. Ungar, *J. Mater. Chem.* **2008**, *18*, 2953-2961. [↑](#endnote-ref-5)
